# Potentiality of Sustainable Maize Production under Rainfed Conditions in the Tropics by Triggering Agro-Physio-Biochemical Traits Ascertained from a Greenhouse

**DOI:** 10.3390/plants12244192

**Published:** 2023-12-18

**Authors:** Md. Samim Hossain Molla, Orawan Kumdee, Arunee Wongkaew, Phanuphong Khongchiu, Nattaporn Worathongchai, Md. Robiul Alam, Abdullah-Al Mahmud, Sutkhet Nakasathien

**Affiliations:** 1 Department of Agronomy, Faculty of Agriculture, Kasetsart University, Bangkok 10900, Thailand; mdsamimhossain.m@ku.tharunee.wo@ku.ac.th (A.W.); 2 On-Farm Research Division, Bangladesh Agricultural Research Institute, Gazipur 1701, Bangladesh; robiula_2013@yahoo.com (M.R.A.); mahmud.tcrc@gmail.com (A.-A.M.); 3 Agricultural Research and Technology Transfer Center, Faculty of Agriculture, Kasetsart University, Bangkok 10900, Thailand; fagrowk@ku.ac.th; 4 Expert Center of Innovative Agriculture, Thailand Institute of Scientific and Technological Research, Pathum Thani 12120, Thailand; phanuphong@tistr.or.th; 5 National Corn and Sorghum Research Center, Faculty of Agriculture, Kasetsart University, Nakhon Ratchasima 30320, Thailand; fagrnow@ku.ac.th

**Keywords:** C absorption, energy efficiency, ethephon, path diagram, profitability, proline, rainfed maize, sustainability, tropics

## Abstract

A major portion of maize is produced under rainfed conditions in the tropics with relatively poor yield because of the unpredictable and irregular distribution of seasonal rainfall, as well as a decline in pre-rainy season rainfall due to climate change, so identification of sustainable production options is utmost needed. Thus, the present studies were conducted in a greenhouse (GH) to ascertain the water stress-tolerant traits of maize and at the field level in the tropical environment of Thailand to see the stimulating possibility of the ascertained traits in a locally popular cultivar using ethephon. Depending on tolerance level, three maize genotypes (Suwan 2301 > Suwan 4452 > S 7328) were tested under different water conditions—well-watered, short-term, and long-term water stress—in the GH. At the field level, the locally popular maize cultivar Suwan 5819 was examined with six ethephon levels (doses in g a.i. ha^−1^ of ethephon, i.e., T1, 281 at V6 stage; T2, 281 at V6 + 281 at V10 stage; T3, 281 at V10 stage; T4, 562 at V6 stage; T5, 562 at V6 + 562 at V10 stage; T6, 562 at V10 stage) against no ethephon application (T0) under rainfed conditions. Maize suffered from the scarcity of sufficient rainfall during 26–39 days after planting (DAP) and 43–63 DAP in the field. The yield index (YI) was identified from biplot analysis as one of the suitable standards for drought tolerance checks for maize at GH as well as at field level in the tropics. The YI value of observed agro-physio-biochemical traits of maize in GH showed that relative water content (RWC, 1.23), stem base diameter (SBD, 1.21), total soluble sugar (TSS, 1.15), proline (Pr, 1.13), aboveground plant biomass (APB, 1.13), root weight (RW, 1.13), relative growth rate (RGR, 1.15), specific leaf weight (SLW, 1.12), and net assimilation rate (NAR, 1.08) were the most desirable. Efforts were made to stimulate these traits under water stress at the field level. Ethephon application as T1 helped to gain higher kernel yield (KY) (5.26 t ha^−1^) with the support of higher RWC (90.38%), proline (24.79 µmol g^−1^ FW), TSS (1629 mg g^−1^ FW), SBD (24.49 mm), APB (271.34 g plant^−1^), SLW (51.71 g m^−2^), RGR (25.26 mg plant^−1^ day^−1^), and NAR (0.91 mg cm^−2^ day^−1^) compared to others, especially no ethephon application. Furthermore, the attributes SLW, SBD, Pr, heat utilization efficiency (HUE), 100-kernel weight, TSS, electrolyte leakage, and lodging percentage showed a substantial direct effect and significant correlation with KY. Aside from higher KY, ethephon application as T1 tactics resulted in higher values of energy efficiency (1.66), HUE (2.99 kg ha^−1^ °C days^−1^), gross margin (682.02 USD ha^−1^), MBCR (3.32), and C absorption (6.19 t C ha^−1^), indicating that this practice may be a good option for maize sustainable production under rainfed conditions.

## 1. Introduction

Rainfed agriculture is widespread throughout the world, including tropical regions, with around 80% of cropland under rainfed cultivation [1]. Maize (*Zea mays*) is a major food, forage, and fuel crop that is grown on about 207 million hectares of land in over 110 countries [2], with more than 80% of the area being rainfed [3]. As a tropical country, Thailand has a majority area of maize under a rainfed system [4], about 78% [5]. Rainfed agriculture is mostly practiced due to a lack of access to irrigation or high-cost minimization [6]. Groundwater allocation for agriculture should be reduced in the long run in response to global climate change to prevent aquifer decline caused by unsustainable water withdrawals [7,8]. Climate change consequences, particularly the increasing frequency and intensity of extreme hydro-meteorological events, will put global water management systems at risk [9,10]. Long-term rainfall patterns appear to be somewhat favorable for rainfed maize production in Thailand. As projected by [11], the rainfall has increased in Thailand during the last decade. Several recent studies have predicted a rise in the frequency and size of extreme rainfall in the future [12,13,14,15,16]. Khadka et al. [11] projected that in northeast Thailand, the rainfall may increase by about 2–8% during the rainy season but may decrease by 6–11% during the pre-rainy season with twofold more hot days in 2021–2050. In contrast, it was expected that, while overall rainfall may increase, the seasonal distribution may have anomalies, such as high rainfall occurring in a short period of time, triggering a flood [17], and an erroneous distribution or fewer rainy days [11]. El Niño and La Niña also caused unpredictable rainfall [18,19]. Several investigators reported that rainfall is shifting to early winter or spring seasons, which often creates a scarcity of rainwater during the rainy season in Thailand [11,20]. The increase in anthropogenic greenhouse gases (GHGs) has resulted in worldwide climate change [21,22,23], with the northeast region of Thailand seeing the fastest rate of change, posing considerable concerns about drought, heat, and crop productivity [11].

However, rainfed maize is cultivated in Thailand during the rainy season (July–October), which frequently experiences water scarcity due to improper rainfall distribution [11]. Moreover, it is projected to reduce rainfed maize yield during 2021–2050 by up to 44% compared to the baseline normal climate [24] and the production may decline by 4% during 2020–2029 [25]. The water deficit may affect maize productivity in several ways. Maize is greatly impacted by water stress affecting vegetative growth, dry matter production, reproductive process and development, grain yield, and quality [26,27,28,29]. Water stress influences reductions in relative water content, photosynthesis, crop growth rate, and antioxidant and enzymatic activities [30,31,32] and increases the senescence, electrolyte leakage, and reactive oxygen species [33]. Inadequate soil water affects maize metabolic activity, biomass deposits, and photosynthetic rate by decreasing chlorophyll content in leaves, resulting in a decrease in maize yield [34,35,36,37]. Different organic solutes can adapt to drought conditions by changing a variety of physio-biochemical processes, including plant growth, structure, osmotic potential of plant tissue, and antioxidant defenses [38,39,40]. The amount of yield loss also depends on the length and intensity of the water stress as well as the phenological stage of the crop that is impacted [41,42,43,44]. In accordance with several studies, proline, total soluble sugar, root–shoot ratio, relative water levels, and other factors are crucial for determining how well maize withstands water stress [45,46,47,48,49]. But these may vary under different environments, in the tropical and temperate regions, which need to be verified. Again, drought stress is unpredictable since it can occur at any stage of the plant’s life cycle [50], and drought during the seedling stage is just as important as at the flowering stage of maize [51]. It is possible to find inbred lines and responsible characteristics that are drought-tolerant and can thrive during later growth stages by screening maize cultivars while they are still in the seedling stage [52].

Under the changing climate, to make rainfed maize cultivation in the tropics sustainable, suitable options are required. Several scientists reported that ethephon (2-chloroethylphosphonic acid), a novel plant growth regulator, has some special potential to stand maize plants against water stress conditions [53,54,55]. As an alternative, plant growth retardants could reduce the amount of water used for early-season crops by lowering LAI, which would allow for longer water availability for crucial reproductive activities during drought stress [55,56,57,58]. Ethylene signaling regulates plant growth [59,60], senescence [53,61], and proline production in plants under optimum or stress conditions [59,62]. Total soluble sugar can act as an osmoregulatory solute under water stress conditions. By raising internal plant solute levels, a variety of organic compatible solutes applied either prior to or during environmental stress may protect plants from damage [63,64,65,66,67]. In contrast, one of the most catastrophic climate extremes is extreme rainfall [11,14,16,68]. Extreme rainfall in a certain short period, especially during the later growth stage, i.e., kernel filling stage of maize, may increase the lodging percentage of maize plant and can significantly hamper kernel growth and ultimately kernel yield [54,55,69]. Scientists found that ethephon enhances stem strength by increasing stem dry matter concentration and stem width and reducing plant height and leaf area, which helps plants stand against lodging [54,55,69,70,71,72,73,74]. However, it is imperative to investigate the effectiveness of ethephon for maize production under rainfed conditions, and in this regard, stress tolerance indices may be helpful methods for identifying the stress tolerance potential of crop genotypes or technologies [35].

Energy inputs in agriculture have rapidly increased, providing numerous benefits to farmers, but they also have a negative impact on the environment [75] by degrading water and land resources and significantly contributing to global warming through increased GHGs [76,77,78,79,80,81]. In light of sustainability, it is crucial to evaluate the energy usage and carbon absorption by maize in the rainfed system. Researchers have also urged conducting a farming system analysis for various crops, including resource use efficiency, crop productivity, economic analysis of a production system, etc. for assessing sustainability [82]. The sustainability of an agricultural production system may be assessed by several indicators. Higher productivity and profitability with environment-friendly techniques may be vital indicators for sustainable maize production in rainfed areas.

However, since agriculture is so dependent on the environment, it is vital to find ways for this sector to adapt to climate change [25]. Plant growth regulators may improve maize’s stress tolerance; however, this varies based on the frequency and concentration of treatment, as well as the stage of plant development. It is critical to establish the appropriate plant application stage and growth regulator dose to maximize the beneficial stress-tolerant characteristics in maize for increased productivity. Therefore, the present study was undertaken to ascertain desirable water stress-tolerant agro-physio-biochemical traits of maize from the greenhouse and to see the potentiality of triggering those traits for sustainable rainfed maize production through proper application tactics of ethephon.

## 2. Results

The results of greenhouse and field experiments are presented separately.

### 2.1. Results of the Greenhouse Experiment

The ANOVA results for the agro-physio-biochemical traits of maize grown in a greenhouse (GH) experiment are shown in Table S1. These results show the independent effects of each genotype and water level, as well as their interactions, on the studied 13 types of traits, i.e., RL3: root length from third (final) sampling at 13 days after watering treatment started (DAWTS); SBD3: stem base diameter at 13 DAWTS; PH3: plant height at 13 DAWTS; LA1, LA2 and LA3: fully expanded green leaf area at 5, 9 and 13 DAWTS respectively; APB3: aboveground plant dry biomass at 13 DAWTS; RW3: root weight at 13 DAWTS; TPB1, TPB2, TPB3: total plant dry biomass at 5, 9 and 13 DAWTS respectively; RGR-T1, RGR-T2, RGR-T3: relative growth rate based on total plant biomass at 5–9, 9–13 and 5–13 DAWTS, respectively; NAR-T1, NAR-T2, NAR-T3: net assimilation rate based on total plant biomass at 5–9, 9–13 and 5–13 DAWTS, respectively; SPAD: SPAD value for leaf greenness at 13 DAWTS; RWC3: relative water content at 13 DAWTS; Pr3: proline at 13 DAWTS; and TSS3: total soluble sugar at 13 DAWTS. The effects of water levels as well as genotypes were significant for all traits. The effect of the interaction between water level and genotype was significant for maximum traits except RL3, TPB2, and SPAD3. However, in the greenhouse experiment, the stress tolerance of several characteristics of maize genotypes was evaluated based on total biomass production during the vegetative stage, which was the ultimate yield. As such, total biomass output with growth-dynamics trends is provided in little detail here, and other characteristics are demonstrated using stress tolerance indicators.

#### 2.1.1. Relative Soil Moisture Dynamics in GH Experiment

**Figure 1:**
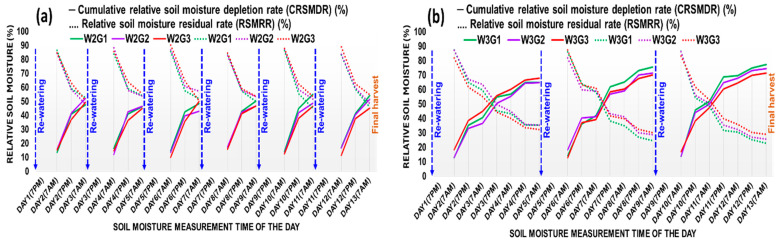
Relative soil moisture dynamics during the 13 days of watering treatment period in (**a**) short-term water stress, W2: 2 days of watering interval and (**b**) long-term water stress, W3: 4 days of watering interval. G1: Suwan 2301, G2: Suwan 4452, and G3: S 7328.

The volumetric soil moisture dynamics for 13 days (16–28 days after planting) for watering treatments imposed in short-term water-stressed (W2) and long-term water-stressed (W3) treatments are presented in Figure 1a,b, respectively. In the case of W2 pots, the cumulative relative soil moisture depletion rate (CRSMDR) was relatively higher and vice versa, the relative soil moisture residual rate (RSMRR) was lower under G2 during the first five days, and afterward, it was changed, and the said trend was observed under G1 up to 13 days. After 13 days of watering treatment in W2, the CRSMDR was 4.84% and 16.69% higher and the RSMRR was 5.66% and 19.61% lower in G1 compared to G2 and G3, respectively. In W3 pots, the CRSMDR and the RSMRR were more acute than W2. During the first five days, the CRSMDR trend was higher and the RSMRR trend was lower in G3, but afterward, up to 13 days, higher CRSMDR and lower RSMRR were found in G1. After 13 days of watering treatment in W3, the CRSMDR was 3.66% and 7.87% higher and the RSMRR was 12.33% and 26.87% lower in G1 compared to G2 and G3, respectively.

#### 2.1.2. Plant Growth Dynamics in GH Experiment

**Figure 2:**
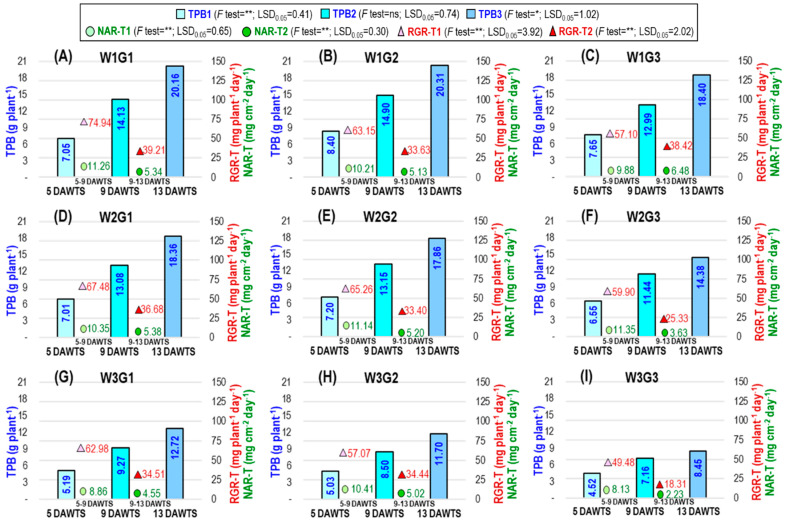
Interaction effect of water levels and genotypes on TPB (total plant biomass), RGR-T (relative growth rate based on total plant biomass), and NAR-T (net assimilation rate based on total plant biomass). (**A**–**I**) represent the interaction treatments W1G1, W1G2, W1G3, W2G1, W2G2, W2G3, W3G1, W3G2, and W3G3, respectively. DAWTS: days after watering treatments started; 1, 2, and 3 stage: 5, 9, and 13 DAWTS; V6: vegetative 6-leaves stage; V10: vegetative 10-leaves stage; VT: vegetative tasseling stage; W1: watering every day; W2: 2 days of watering interval; W3: 4 days of watering interval; G1: Suwan 2301, G2: Suwan 4452, and G3: S 7328; * and **: significant at *p* < 0.05 and *p* < 0.01 levels, respectively; ns: nonsignificant.

The dry weight of each plant’s roots and shoots made up the total plant biomass (TPB). The interaction between water level and genotype had a significant impact on the TPB at 5 and 13 DAWTS except 9 DAWTS (TPB1, TPB3, and TPB2, respectively), the net assimilation rate based on TPB (NAR-T) during 5–9 DAWTS and 9–13 DAWTS, and the relative growth rate based on TPB (RGR-T) during 5–9 DAWTS and 9–13 DAWTS (Figure 2).

The overall TPB reduced more under W3 than under W2 at all DAWTS. At 13 DAWTS, W2 caused the TPB3 to drop by 8.93%, 12.06%, and 21.85%, while W3 caused the TPB3 to decrease by 36.90%, 42.39%, and 54.08% in G1, G2, and G3, respectively. In G2 and G3, respectively, the decline in TBP3 under W3 was 14.88% and 48.78% greater than in G1. Compared to the 5–9 DAWTS period, the NAR-T was more impacted by W2 and W3 during the 9–13 DAWTS period. W2 exhibited no detrimental effects on G1 or G2 NAR-T throughout the 9–13 DAWTS period, but it had a substantial adverse effect on G3 (43.98% lowered). Under W3, NAR-T was reduced more in G3 (65.59%) and was more than threefold the value in G1. The RGR-T was more severely affected by W3 than W2 in all genotypes. Higher RGR-T was found in G1, which was identical to G2 and was 46.94% decreased in G3 compared to G1.

#### 2.1.3. Principal Component Analysis (PCA) and Correlation Coefficients of Drought Tolerance Indices in GH

In accordance with the genotypes’ total plant biomass, drought tolerance indices were calculated under long-term water stress conditions for three genotypes in the GH (Table 1). Plant biomass under long-term water stress (YS) is reduced in comparison to plant biomass under non-stress conditions (YP). In the current experiment, the biomass yields for the Suwan 2301, Suwan 4452, and S 7328 genotypes under long-term water stress conditions were 36.90%, 42.39%, and 54.08% lower than those under well-watered conditions. The correlation coefficients between YP, YS, and other quantitative measures of drought tolerance were studied to ascertain the most suitable drought tolerance standards (Table 2). The findings revealed that ATI did not substantially connect with Ys, whereas MP, MRP, GMP, REI, STI, MSSIk1, MSTIk2, HARM, YI, RDI, DI, GM, SNPI, and DTE were positively and significantly correlated with Ys. Significant association with Ys demonstrates that these indices are good predictors of drought resistance; however, the absence of correlation between Ys and the ATI index demonstrates that this index is not a useful predictor of drought tolerance in maize. Except for ATI, drought indices were highly correlated with Yp and others.

**Figure 3:**
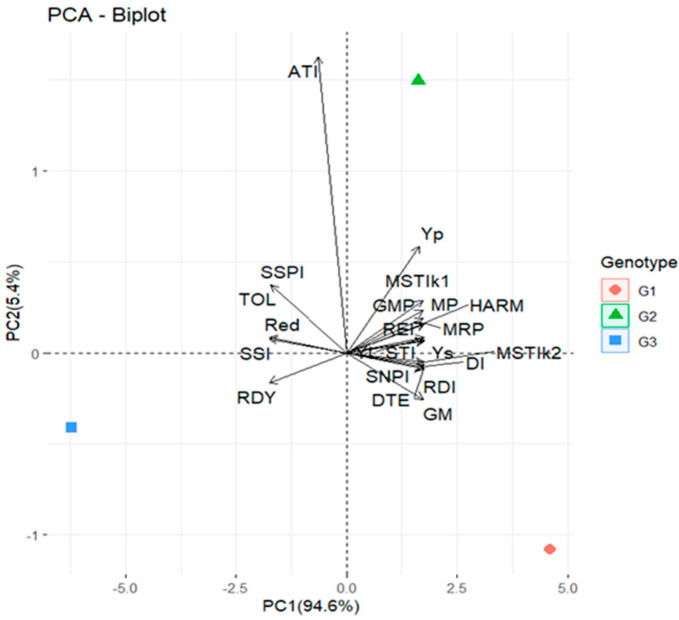
The biplot diagram of principal component analysis (PCA) of three genotypes of maize according to mean measured drought tolerance indices together with mean total plant biomass under long-term water stress (Ys) and well-watered (Yp) situations. MP, mean productivity; MRP, mean relative performance; SSI, stress susceptibility index; TOL, tolerance index; GMP, geometric mean productivity; REI, relative efficiency index; STI, stress tolerance index; MSTIk1, modified stress tolerance index 1; MSTIk2, modified stress tolerance index 2; HARM, harmonic mean of yield; YI, yield index; Red, reduction; RDI, relative drought index; DI, drought resistance index; GM, golden mean; ATI, abiotic tolerance index; SSPI, stress susceptibility percentage index; SNPI, stress/non-stress production index; RDY, relative decrease in yield; DTE, drought tolerance efficiency; G1, Suwan 2301; G2, Suwan 4452; G3, S 7328.

A useful approach to determining drought tolerance could include selection based on a combination of drought tolerance indices. For treatment combinations (genotypes and stress indices), principal component analysis (PCA) was employed. The biplot’s first two axes fully explained most variation. The first axis (PCA 1 = 94.6%) and the second axis (PCA 2 = 5.4%), respectively, account for the most variation (Figure 3). Although Suwan 4452 exhibited good plant biomass productivity (under water stress, Ys), the biplot estimates that, in comparison to the other cultivars, its biomass was severely impacted by drought (high ATI and also TOL). On the other hand, Suwan 2301’s high YI, STI, GM, MRP, REI, MSTIk2, HARM, RDI, DI, SNPI, and DTE indicates that the impact of water stress on biomass production is minimal.

The cosine of the angle between the vectors of any two indices represents the correlation coefficient. As an illustration, r = Cos180° = −1, Cos 0° = 1, and Cos 90° = 0 [83]. The angles between Ys, Yp, MP, MRP, GMP, REI, STI, MSSIk1, MSTIk2, HARM, YI, RDI, DI, GM, SNPI, and DTE in the biplot diagram (Figure 3) are acute, indicating a positive association, which is validated by correlation analysis (Table 2). The PCA1 and PCA2 axes, which account for 100% of total variation, primarily differentiate the indices into groups. In group 1, the PCs axis separated the indices Ys, Yp, MP, MRP, GMP, REI, STI, MSSIk1, MSTIk2, HARM, YI, RDI, DI, GM, SNPI and DTE. The SSPI, TOL, Red, SSI, and RDY were classified as group 2, while ATI was classified as group 3. However, higher but negative ATI indicates that Suwan 4452 is more susceptible to water stress. The angles between YI and STI with REI, MRP, MP, GMP, SNPI, DTE, RDI, HARM, MSTIk1, and MSTIk2 were acute, which indicates their suitability for drought tolerance measurement. However, at the field level, there was no control treatment for water, so only YI was selected and further used for index measurement to compare different parameters at both the greenhouse and field levels.

#### 2.1.4. Selection of Traits Based on Yield Index (YI) in GH

**Figure 4:**
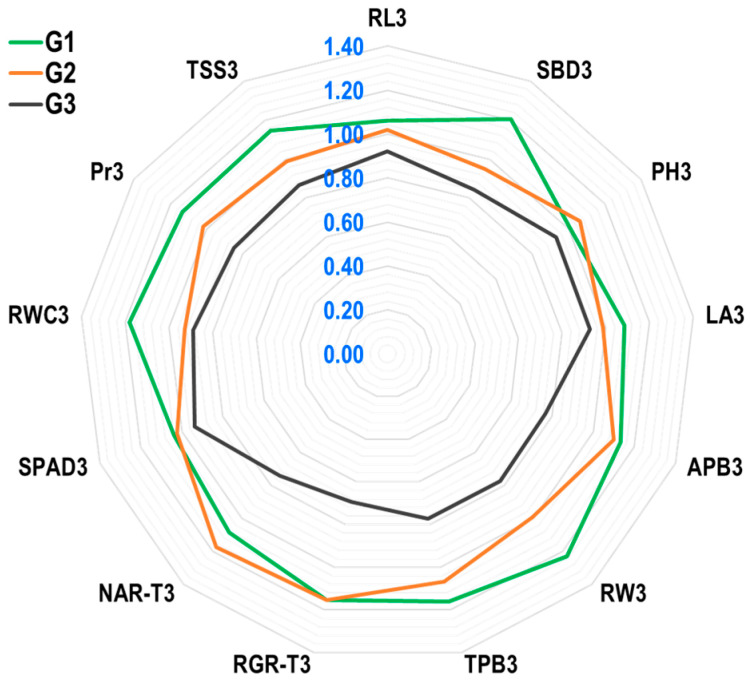
Radar plot showing yield index (YI) of 13 characteristics of 3 maize genotypes in respect to tolerance against long-term water stress in greenhouse. The numbers shown in blue in the graphic indicate the whole YI scale. G1, Suwan 2301; G2, Suwan 4452; G3, S 7328; RL3: root length from 3rd (final) sampling at 13 days after watering treatment started (DAWTS); SBD3: stem base diameter at 13 DAWTS; PH3: plant height at 13 DAWTS; LA3: leaf area at 13 DAWTS; APB3: aboveground plant dry biomass at 13 DAWTS; RW3: root weight at 13 DAWTS; TPB3: total plant dry biomass at 13 DAWTS; RGR-T3: relative growth rate based on total plant biomass at 5–13 days during watering treatment; NAR-T3: net assimilation rate based on total plant biomass at 5–13 days during watering treatment; SPAD3: SPAD value for leaf greenness at 13 DAWTS; RWC3: relative water content at 13 DAWTS; Pr3: proline at 13 DAWTS; TSS3: total soluble sugar at 13 DAWTS.

The radar plot results (Figure 4) showed that the genotype Suwan 2301 (G1) has excellent drought tolerance due to a high YI for traits SBD3, LA3, APB3, RW3, TPB3, RGR3, SPAD3, RWC3, Pr3, TSS3, and RL3. Despite the fact that the YI value for NAR-T3 in G1 was moderate, it was greater than one, which is significant and acceptable. In G1, the YI value for PH3 was lower than in G2. Suwan 4452 had comparatively high YI values for PH3 and NAR-T3, whereas SPAD3 and RGR-T3 were less affected by water stress, with lower YI values than Suwan 2301. The genotype S 7328 (G3) had the lowest Yi values for all traits, with values less than one for RL3, SBD3, PH3, LA3, APB3, RW3, TPB3, RGR3, NAR-T3, SPAD3, RWC3, Pr3, and TSS3. Suwan 2301, therefore, demonstrated a higher tolerance to water stress by showing significant and high YI values in RW3 (1.23), SBD3 (1.21), RWC3 (1.18), TPB3 (1.16), RGR-T3 (1.15), TSS3 (1.15), Pr3 (1.13), APB3 (1.13), NAR-T3 (1.08), RL3 (1.06), and SPAD3 (1.04), which may be desirable traits for field-level maize production under rainfed conditions.

### 2.2. Results of the Field Experiment

The ANOVA results for the agronomic and agro-physio-biochemical traits of maize grown in the field experiment are shown in Tables S2 and S3, respectively. These results show the effects of ethephon application tactics (EAT) on the 23 types of studied parameters, i.e., PH-PM: plant height at physiological maturity (PM); EH-PM: ear height at PM; EH:PH: ear height and plant height ratio; SBD-VT: stem base diameter at vegetative tasseling stage (VT); LA-V6, LA-V10, LA-VT, and LA-R3: leaf area at 6-leaves stage (V6), at 10-leaves stage (V10), at VT, and at reproductive milking stage (R3), respectively; APB-V6, APB-V10, APB-VT, and APB-R3: aboveground plant biomass at V6, V10, VT, and R3, respectively; KNP: kernel number per plant; 100-KW: 100-kernel weight; KY: kernel yield; SLW-VT: specific leaf weight at VT; LP-PM: lodging percentage at PM; RGRA-V6V10, RGRA-V10VT, and RGRA-V6R3: relative growth rate based on aboveground plant biomass during V6 to V10, V10 to VT, and V6 to R3, respectively; NARA-V6V10, NARA-V10VT, and NARA-V6R3: net assimilation rate based on aboveground plant biomass during V6 to V10, V10 to VT, and V6 to R3, respectively; AGDDs: accumulated growing degree days; HUE: heat use efficiency; RWC-VT: relative water content at VT; SPAD-VT: SPAD value of leaf greenness at VT; RSR-VTR3: relative senescence rate during VT to R3; EL-VT: electrolyte leakage at VT; C: carbon; TSS-VT: total soluble sugar at VT; Pr-VT: proline content at VT. The effects of ethephon application tactics (EAT) were significant for most of the agronomic traits except SBD-VT, LA-V6, APB-V6, APB-V10, and KNP. The effects of EAT were also significant for maximum agro-physio-biochemical traits except RGRA-V6V10, RGRA-V10VT, NARA-V6V10, AGDDs, RWC-VT, and SPAD-VT.

#### 2.2.1. Environmental Conditions of the Field Experimental Location

**Figure 5:**
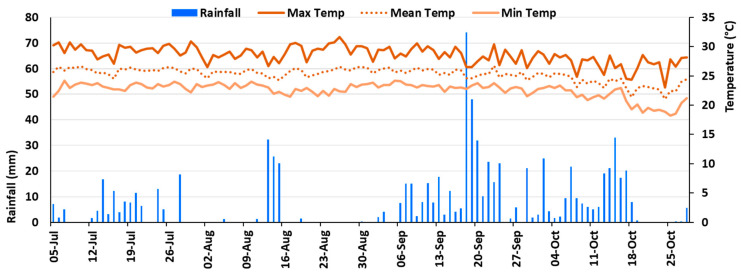
Daily temperature (°C, maximum, mean, minimum) and rainfall (mm, total) during the experimental period in 2020 at Pakchong, Nakhon Ratchasima, Thailand.

The long-term data showed that climate change impacted the weather of Nakhon Ratchasima province of Thailand (Figure 5 and Figure S1). The mean annual temperature is on an increasing trend, and it has increased by about 0.8 °C per decade since the 1950s. The annual rainfall range is 893.36 mm to 1603.81 mm from 1901 to 2021, where the maximum rainfall occurred in 2017 and the minimum occurred in 1979. It was found that December and January are relatively cool, and March to May are relatively the hottest. On the other hand, the long-term data indicated that the mean temperature in January 2020 was approximately 25 °C, whereas the current data indicated that it was approximately 23 °C (Figure 5). However, the warmer months continued to get hotter, and it was discovered that the maximum temperature in April 2020 was approximately 41 °C, although long-term statistics indicated that it was approximately 36 °C. When it came to rainfall, the long-term data indicated that the highest amounts happened in September followed by August, while the lowest amounts were in January and December. In contrast, the current data revealed that there was no rainfall in January, February, November, and December.

According to Figure 5, there was approximately 813 mm of rainfall over the trial period, with the maximum, mean, and lowest temperatures being recorded at 23.04–31.62, 21.04–26.72, and 18.26–24.23 °C, respectively. According to [84,85], maize typically requires 650 mm of water as a whole (with a range of 500–800 mm) in well-watered conditions. Therefore, the amount of rainfall during the current crop period would have been adequate; unfortunately, the distribution of the rainfall was not proper. From the second week of September to the third week of October, there was the most rainfall, with the highest one-day total of almost 74 mm in the third week of September. Contrarily, there were only two days of very little rainfall (total of 2.49 mm) during the 24 to 38 DAP (15 days), and only four days of very little rainfall (total of 7.76 mm) during the 42 to 62 DAP (21 days). However, in the reddish-brown lateritic soil in Nakhon Ratchasima, Thailand, which was also the current experimental location [86,87], approximately 40 mm of water needs to be supplied each week for maize growth and development. Thus, during the aforementioned initial two weeks (24 to 38 DAP) and the second three weeks (42 to 62 DAP) of rainfall shortage, the crop experienced water scarcity. These times corresponded to the stages of maize growth, 5 to 11 leaves, and pre-flowering to bracketing flowering, respectively.

#### 2.2.2. Plant Growth Dynamics in the Field Experiment

**Figure 6:**
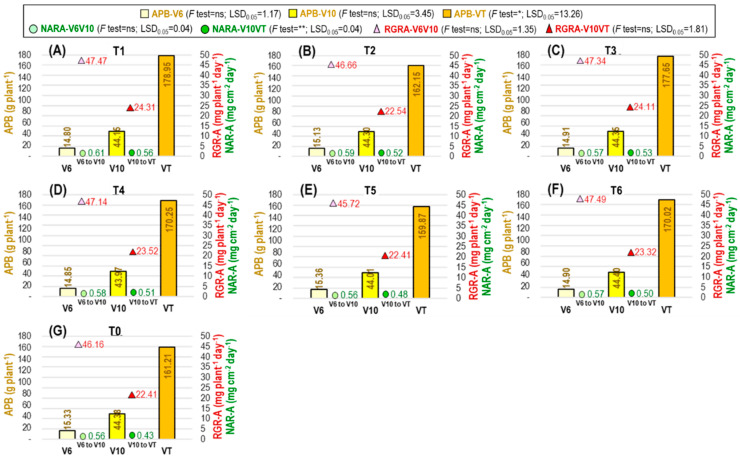
Effect of ethephon tactics on APB (aboveground plant biomass) and RGR-A (relative growth rate based on aboveground plant biomass). (**A**–**G**) represent the ethephon treatments T1 (ethephon@ 281 at V6 stage), T2 (ethephon@ 281 at V6 + 281 at V10 stage), T3 (ethephon@ 281 at V10 stage), T4 (ethephon@ 562 at V6 stage), T5 (ethephon@ 562 at V6 + 562 at V10 stage), T6 (ethephon@ 562 at V10 stage), and T0 (no ethephon application), respectively. V6: vegetative 6-leaves stage; V10: vegetative 10-leaves stage; VT: vegetative tasseling stage; * and **: significant at *p* < 0.05 and *p* < 0.01 levels, respectively; ns: nonsignificant.

The aboveground plant biomass (APB) did not differ significantly before ethephon application at the V6 (APB-V6) stage or after application at the V10 (APB-V10) stage, but differed significantly among the treatments at the VT (APB-VT) stage (Figure 6) and R3 (APB-R3) stage (Table 3). At the VT stage, the APB was more influenced by ethephon application T1 tactics, which was 9.91% higher than no ethephon application (T0). At the R3 stage, the highest APB was obtained from T1, which was 7.45% higher than T0. Identically minimum APB at the R3 stage was obtained from T4, T5, and T6 treatments.

The net assimilation rate based on APB (NARA) was significantly affected by ethephon application tactics during the V10 to VT (NARA-V10VT) period (Figure 6) and the V6 to R3 (NARA-V6R3) period (Table 4). During the V10 to VT stage, the maximum NARA was found in T1, which was 23.21% higher than T0. The highest NARA between V6 to R3 stage was also found in T1, and it was 19.78% higher than T0.

The relative growth rate based on APB (RGRA) differed significantly only between V6 to R3 (RGRA-V6R3) (Table 4) but not differ for V6 toV10 (RGRA-V6V10) or V10 to VT (RGRA-V10VT) (Figure 6). During the V6 to R3 stage, the lowest RGRA-V6R3 was observed in T0, and it was 4.04% and 3.62% higher in T1 and T3, respectively.

#### 2.2.3. Other Agronomic Traits of Maize as Influenced by Ethephon Application Tactics

PH-PM: The plant height of maize at the physiological maturity stage was significantly decreased due to ethephon application at all tactics (Table 3). The maximum plant height was measured in T0, and it was 5.97%, 13.06%, 5.67%, 10.48%, 21.80%, and 9.57% lower in T1, T2, T3, T4, T5, and T6 treatments, respectively. Application of a higher dosage of ethephon was more detrimental for plant height, especially when applied at both V6 and V10 stages.

EH-PM: The ear height was significantly decreased due to ethephon application at all tactics (Table 3). The highest ear height was found in T0, and it was 26.32%, 34.79%, 26.89%, 33.46%, 44.73%, and 34.67% lower in T1, T2, T3, T4, T5, and T6 treatments, respectively. The ear height was decreased more under application at both V6 and V10 stages with a higher dosage of ethephon.

EH:PH ratio: Under the present study, it was found that the maximum ratio (48.68%) was in T0, and the minimum was in T5 (34.41%), which was treated with higher dosages of ethephon in two growth stages of the maize plant. The EH:PH ratio of T1, T2, T3, and T4 was 38.15%, 36.52%, 37.73%, and 36.18%, respectively.

SBD-VT: The stem base diameter is related to maize plant strength against lodging and dry matter reserve. The SBD-VT was a little bit higher in T1 treatment and lower in T5 treatment, but those were nonsignificant.

LA-V6, V10, VT, R3: The leaf area at V10 (LA-V10), LA-VT, and LA-R3 was higher in T0, and the LA at all stages decreased due to ethephon application. The higher dosages decreased LA more. Moderate and balanced LA was found in T1 and T3 treatments.

KNP: The kernel number per plant is an important determinant for kernel yield. Though it was nonsignificant, slightly higher KNP was found in the T1 and T4 treatments and lower in the T0 treatment.

100-KW: The ethephon application triggers the kernel weight significantly. The lowest 100-KW was found in the T0 treatment, and it was increased by 17.39%, 13.95%, 16.64%, 15.71%, 11.86%, and 15.57%, in T1, T2, T3, T4, T5, and T6 treatments, respectively, compared to T0.

KY: Kernel yield of maize is the ultimate target, and it was significantly influenced by ethephon application tactics. The identically higher kernel yield was obtained from T1, T3, and T2 treatment (Table 3). The lowest kernel yield was in the T0 treatment, and it was increased due to the ethephon application. Due to the application of ethephon as T1, T2, T3, T4, T5, and T6, the KY was increased by 30.85%, 23.13%, 27.11%, 13.43%, 5.47%, and 12.19%, respectively.

#### 2.2.4. Agro-Physiological Traits of Maize as Influenced by Ethephon Application Tactics

SLW-VT: The specific leaf weight might be altered under ethephon application tactics, and it was found that after the application of ethephon, the SLW significantly increased at the vegetative tasseling stage. The SLW was higher and identical in all ethephon application tactics, where T1 was influenced slightly more to increase the SLW (22.51%) compared to T0. (Table 4).

LP-PM: The lodging percentage of the plant was counted at the physiological maturity stage (LP-PM) and it was observed that ethephon application tactics significantly reduced the lodging rate of the maize plant in the field (Table 4). The minimum lodging percentage was found in the T2 and T5 treatments. Compared to no ethephon application tactics (T0), the lodging percentage of plants was reduced by 79.68%, 85.80%, 74.43%, 79.34%, 87.28%, and 74.76% in T1, T2, T3, T4, T5, and T6, respectively.

#### 2.2.5. Physio-Biochemical Traits of Maize as Influenced by Ethephon Application Tactics

RWC-VT: For maize plants, the vegetative tasseling stage is crucial for the assimilation production and growth of reproductive organs, both of which may be impacted by the plant’s water availability. However, at the vegetative tasseling stage, the relative water content (RWC-VT) was measured, and a significant variation was found among the treatments (Table 4). This might be due to the shortage of rainwater during that period (Figure 6). Nonetheless, it was found that the RWC-VT was more in T3, which was identical with T1, T2, T3, T4, T5, and T6. The lowest RWC-VT was measured in T0, and it was 9.37% and 6.15% lower than T3 and T1, respectively.

SPAD-VT: The SPAD value for leaf greenness at the vegetative tasseling stage was nonsignificant between ethephon application or none. However, the SPAD-VT value was slightly higher in the ethephon-treated plants, which might be due to higher leaf thickness in these treatments, as the specific leaf weight was higher in these plants.

RSR-VTR3: The relative senescence rate of maize leaves was measured during the vegetative tasseling stage to the reproductive milking stage period (RSR-VTR3). The RSR-VTR3 was more pronounced in T5 followed by T4 and T2, where it was identically lower in T0, T1, T3, and T6.

EL-VT: The electrolyte leakage may increase under stress conditions and may alter under different management systems. However, it was found that ethephon application tactics significantly reduced the electrolyte leakage at the vegetative tasseling stage (EL-VT) compared to no ethephon application (Table 5). The EL-VT was lowered by 16.81%, 18.68%, 16.14%, 13.43%, 14.27%, and 15.12% in T1, T2, T3, T4, T5, and T6, respectively, compared to T0.

TSS-VT: The total soluble sugar at the vegetative tasseling stage (TSS-VT) was significantly increased due to ethephon application (Table 4). Though the TSS-VT was identically higher in all ethephon-treated plants, a higher increment was found in T1 (21.02%) compared to T0.

Pr-VT: The proline content in maize leaves at the vegetative tasseling stage (Pr-VT) was influenced significantly by ethephon application (Table 4). Identically higher Pr-VT was found in all ethephon-treated plants compared to no ethephon application. The Pr-VT was increased by 54.26%, 49.22%, 54.01%, 50.28%, 48.72%, and 49.53% in T1, T2, T3, T4, T5, and T6, respectively, compared to T0.

#### 2.2.6. Analysis of Correlation and Path Coefficient Separately for Agronomic, Agro-Physiological, and Physio-Biochemical Traits

**Figure 7:**
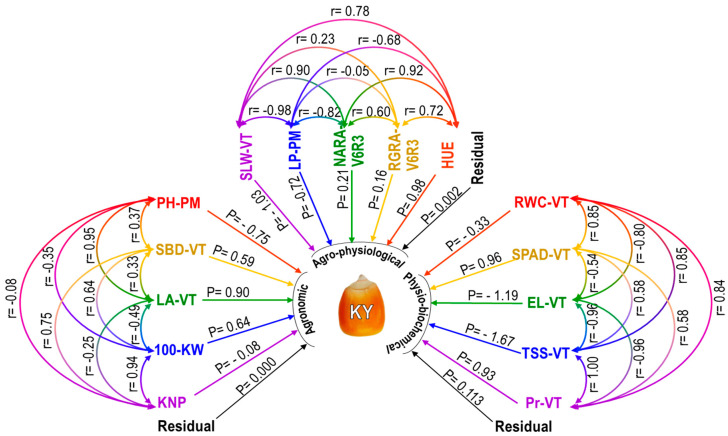
Path diagram showing the relationships between several agronomic (**left side**), agro-physiological (**upper side**), and physio-biochemical (**right side**) characteristics of maize under rainfed conditions at the field level. The direct impact (P) on kernel yield (KY) is indicated by a single arrow, while the correlation coefficient (r) between attributes is indicated by a double arrow. V6, vegetative 6-leaves stage; VT, vegetative tasseling stage; R3, reproductive phase 3 of maize plant; PM, physiological maturity stage; KNP, kernel number per plant; 100-KW, 100-kernel weight; LA-VT, leaf area at VT; SBD-VT, stem base diameter at VT; PH-PM, plant height at PM; SLW-VT, specific leaf weight at VT; LP-PM, lodging percentage at PM; NARA-V6R3, net assimilation rate based on aboveground plant biomass during V6 to R3; RGRA-V6R3, relative growth rate based on aboveground plant biomass during V6 to R3; HUE, heat use efficiency; RWC-VT, relative water content at VT; SPAD-VT, SPAD value of leaf greenness at VT; EL-VT, electrolyte leakage at VT; TSS-VT, total soluble sugar at VT; Pr-VT, proline content at VT.

Agronomic, agro-physiological, and physio-biochemical parameters were selected and their individual contributions to kernel yield (KY) were assessed by correlation and path coefficient analysis. The correlation analysis of selected five agronomic traits showed that the SBD-VT, KNP, and 100-KW had the highest, second-highest, and third-highest positive and significant correlations with KY, respectively, while PH-PM and LA-VT showed a lower but positive correlation (Table 5). The PH-PM had positive correlations with SBD-VT and LA-VT, but a negative correlation was found with 100-KW and KNP (Figure 7). The SBD-VT had positive and significant correlations with 100-KW and KNP, and also positive with LA-VT. A negative correlation was found between LA-VT and 100-KW and KNP. A strong and positive correlation was observed between 100-KW and KNP.

Based on a correlation analysis of five agro-physiological traits, almost all of the traits (SLW-VT, NAR-V6R3, RGR-V6R3, and HUE) showed a strong positive association with KY, with the exception of LP-PM, which showed a strong negative correlation (Table 5). Figure 7 demonstrated that SLW-VT correlated significantly and favorably with HUE, NARA-V6R3, and RGRA-V6R3. However, it correlated negatively and strongly with LP-PM. The LP-PM had a negative but negligible correlation with RGRA-V6R3 and a strong negative correlation with NARA-V6R3 and HUE. Significant positive correlations were found between the NARA-V6R3, RGRA-V6R3, and HUE. There was a substantial positive correlation between the RGRA-V6R3 and HUE.

The study conducted a correlation analysis on five different physio-biochemical traits. The results indicated that, with the exception of EL-VT, which had a strong negative correlation with KY, almost all of the traits, namely, RWC-VT, SPAD-VT, TSS-VT, and Pr-VT, were positively and significantly connected with KY (Table 5). RWC-VT exhibited a substantial negative relationship with EL-VT, but a significant positive correlation with SPAD-VT, TSS-VT, and Pr-VT, as Figure 7 demonstrates. Significant and positively related to TSS-VT and Pr-VT, but negatively correlated with EL-VT, was the SPAD-VT. Significant negative correlations were seen between the EL-VT, TSS-VT, and Pr-VT. There was a strong positive correlation between the TSS-VT and Pr-VT.

Route coefficient analysis allows one to identify the direct and indirect effects of different parameters on kernel yield because it is essentially a standardized partial regression coefficient. Regarding agronomic traits, KY was highly positively impacted by SBD-VT, LA-VT, and 100-KW, while PH-PM and KNP had somewhat negative direct effects on KY (Figure 7). KNP’s direct effect was negligible because it was less than 0.1. Table 5 indicates that the PH-PM had a favorable indirect influence on KY via SBD-VT and LA-VT, but it had a negative indirect effect on KY via 100-KW, according to the indirect effect of agronomic characteristics. Through LA-VT and 100-KW, the SBD-VT positively impacted KY indirectly. Through PH-PM and 100-KW, the LA-VT had a negative indirect effect on KY; but, through SBD-VT, it had a positive effect. Through PH-PM and SBD-VT, the 100-KW had a positive indirect effect on KY; however, through LA-VT, it had a negative indirect effect. Through SBD-VT and 100-KW, the KNP had positive effects on the KY.

As regards agro-physiological characteristics, SLW-VT and LP-PM showed strong but negative direct effects on KY, while NARA-V6R3, RGRA-V6R3, and HUE had positive direct effects (Figure 7). It was found that SLW-VT had a positive indirect impact via LP-PM, NARA-V6R3, RGRA-V6R3, and HUE in the case of the indirect effect of agro-physiological traits (Table 5). Via NARA-V6R3 and HUE, the LP-PM had a negative indirect effect on KY, whereas SLW-VT exhibited a positive indirect effect. Via HUE, the NARA-V6R3 had a positive indirect effect. Through LA-VT and HUE, the RGRA-V6R3 had a positive impact on the KY. Through SBD-VT and LA-VT, the HUE had positive indirect effects on KY; however, through PH-PM, it had negative indirect effects.

Regarding the physio-biochemical characteristics, KY was positively impacted by the SPAD-VT and Pr-VT, while RWC-VT, EL-VT, and TSS-VT had a negative direct effect on KY (Figure 7). Regarding the physio-biochemical trait indirect impact, it was discovered that RWC-VT had a negative indirect effect via TSS-VT and a positive indirect effect via SPAD-VT, EL-VT, and Pr-VT (Table 5). Through EL-VT and Pr-VT, the SPAD-VT indirectly impacted KY, whereas TSS-VT and RWC-VT had a negative impact. While SPAD-VT and Pr-VT exhibited a negative indirect effect, TSS-VT and RWC-VT had a positive indirect effect from the EL-VT. Through SPAD-VT, EL-VT, and Pr-VT, the TSS-VT had positive effects on the KY; via RWC-VT, it had a negative effect on the KY. Indirect effects of the Pr-VT were found to be negative through 100-KW and PH-PM, and positive through SPAD-VT and EL-VT.

#### 2.2.7. Yield Index (YI) of Agro-Physio-Biochemical Traits of Maize at Field Level

**Figure 8:**
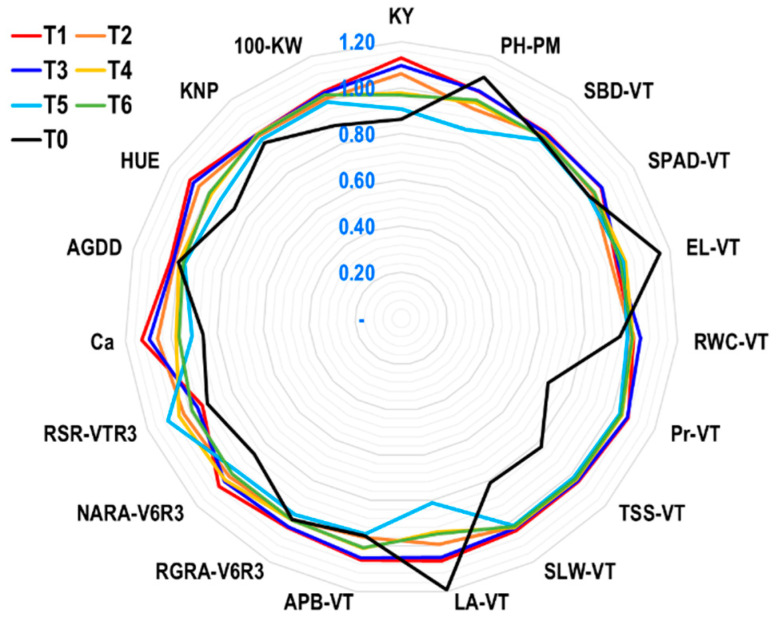
Radar plot showing yield index (YI) of 19 characteristics of maize grown under rainfed conditions. The numbers shown in blue in the graphic indicate the whole YI scale. T1: ethephon @ 281 g at V6 stage; T2: ethephon @ 281 g at V6 + 281 g at V10 stage; T3: ethephon @ 281 g at V10 stage; T4: ethephon @ 562 g at V6 stage; T5: ethephon @ 562 g at V6 + 562 g at V10 stage; T6: ethephon @ 562 g at V10 stage; T0: no ethephon application. V6: vegetative 6-leaves stage; VT: vegetative tasseling stage; R3: reproductive phase 3; PM: physiological maturity stage; PH-PM: plant height at PM; SBD-VT: stem base diameter at VT; SPAD-VT: SPAD value of leaf greenness at VT; EL-VT: electrolyte leakage at VT; RWC-VT: relative water content at VT; Pr-VT: proline content at VT; TSS-VT: total soluble sugar at VT; SLW-VT: specific leaf weight at VT; LA-VT: leaf area at VT; APB-VT: aboveground plant biomass at VT; RGRA-V6R3: relative growth rate based on aboveground plant biomass during V6 to R3; NARA-V6R3: net assimilation rate based on aboveground plant biomass during V6 to R3; RSR-VTR3: relative senescence rate during VT to R3; Ca: carbon absorption; AGDDs: accumulated growing degree days; HUE: heat use efficiency; KNP: kernel number per plant; 100-KW: 100-kernel weight; KY: kernel yield.

The yield index (YI) of different agro-physio-biochemical traits of maize was calculated under different ethephon application tactics (EAT) to determine the capacity of EAT for triggering the tolerance of different traits under rainfed conditions (Figure 8). It was considered that when the YI was ≥ 1.0, the EAT was significant for triggering the tolerance; when the YI was < 1, the EAT was nonsignificant for triggering the tolerance of different traits.

The findings of the radar plot (Figure 8) under the rainfed maize production system demonstrated that, in comparison to other tactics, the ethephon application T1 tactics followed by T3 efficiently triggered the highest number of agro-physio-biochemical features. The T1 exhibits excellent tolerance because it has high and significant values of YI for the following characteristics: SBD-VT, APB-VT, Pr-VT, TSS-VT, RGRA-V6R3, NARA-V6R3, Ca, AGDDs, HUE, KNP, and 100-KW under rainfed conditions. In contrast, the YI values of PH-PM, EL-VT, and RSR-VTR3 were lower under T1, which is also desired at a lower level for more tolerance. However, under T3, the YI value was comparable to T1 for SBD-VT, Pr-VT, TSS-VT, and RGRA-V6R3 and higher for SPAD-VT and RWC-VT, indicating that it was the second-highest tolerance. Except for PH-PM, EL-VT, and LA-VT, all other characteristics had a lower YI value in the T0 treatment. This suggests that this technique is not very effective at triggering the agro-physio-biochemical features of maize at the field level under rainfed conditions.

#### 2.2.8. Sustainability

##### Accumulated Growing Degree Days (AGDDs) and Heat Use Efficiency (HUE)

**Figure 9:**
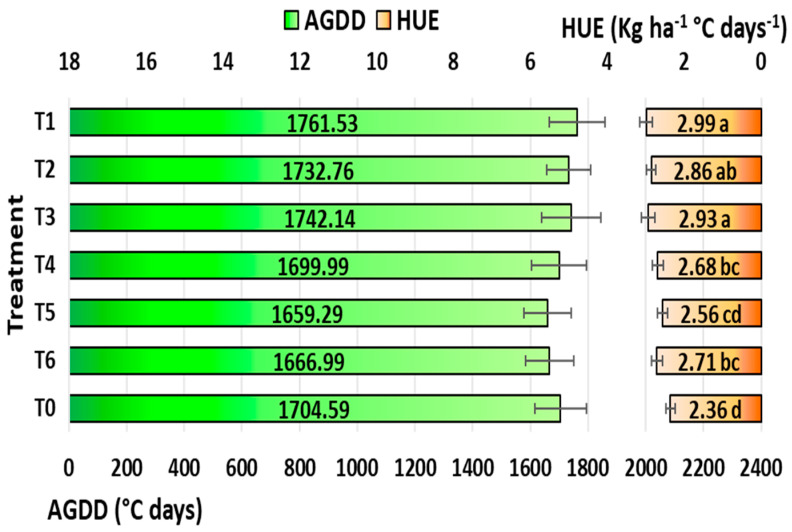
Accumulated growing degree days (AGDDs) on left side and heat use efficiency (HUE) on right side of the graphic as affected by ethephon application tactics in maize under rainfed conditions in the tropics. T1: ethephon @ 281 at V6 stage; T2: ethephon @ 281 at V6 + 281 at V10 stage; T3: ethephon @ 281 at V10 stage; T4: ethephon @ 562 at V6 stage; T5: ethephon @ 562 at V6 + 562 at V10 stage; T6: ethephon @ 562 at V10 stage; T0: no ethephon application. Means within a chart with the same or no letters are not significant at *p* < 0.05 based on LSD test. Horizontal bars are twice the standard deviation.

Crop development rate can be influenced by the accumulation of heat during a crop’s growth season, which is measured by the accumulated growing degree days (AGDDs). The tactics for applying ethephon were not significant for AGDDs (Figure 9). However, slightly more AGDDs were found with T1 tactics (1761.53 °C), and minimal AGDDs were found in T5 treatment (1704.59 °C).

The capacity of a crop to use heat effectively under specific growing conditions is measured by its heat use efficiency. Tactics for applying ethephon have a substantial impact on the HUE (Figure 9). Positive effects of the ethephon application were observed in the HUE, with T1 (2.99 kg ha^−1^ °C days^−1^) having the highest calculated HUE, followed by T3 (2.93 kg ha^−1^ °C days^−1^) and T2 (2.86 kg ha^−1^ °C days^−1^). With 26.69% lower heat usage compared to T1, T0 (2.36 kg ha^−1^ °C days^−1^) was shown to be the least efficient.

##### Carbon Absorption (Ca)

**Figure 10:**
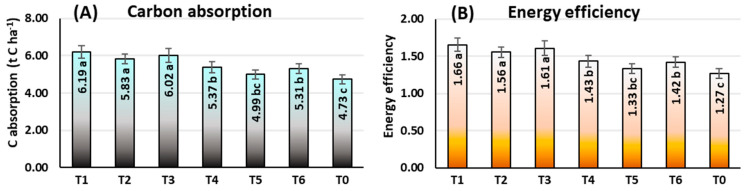
Carbon absorption (**A**) and energy efficiency (**B**) in maize grown under rainfed conditions. T1: ethephon @ 281 at V6 stage; T2: ethephon @ 281 at V6 + 281 at V10 stage; T3: ethephon @ 281 at V10 stage; T4: ethephon @ 562 at V6 stage; T5: ethephon @ 562 at V6 + 562 at V10 stage; T6: ethephon @ 562 at V10 stage; T0: no ethephon application. Means within a chart with the same or no letters are not significant at *p* < 0.05 based on LSD test. Vertical bars are twice the standard deviation.

At the field level, under rainfed conditions, the findings in Figure 10A demonstrate the significant impacts of ethephon application tactics on C intake by maize. At the mature stage (Ca), rainfed maize absorbed the most carbon under T1 tactics (6.19 t C ha^−1^), which was also observed for T3 (6.02 t C ha^−1^) and T2 (5.83 t C ha^−1^) tactics. Plot (T0) with no ethephon applied had the lowest C absorption (4.73 t C ha^−1^). Ethephon was found to be more efficient when used at a single application and at a relatively lower dosage for C absorption. In T1 and T3, respectively, C absorption was estimated to be 30.87% and 27.27% greater than in T0.

##### Energy Efficiency

The ethephon application tactics had significant effects on energy efficiency, and the findings indicated that energy efficiency was comparatively higher at lower ethephon application dosages (Figure 10B). On the other hand, in rainfed conditions, T0 (1.27) had the lowest efficiency, while T1 (1.66), T3 (1.61), and T2 (1.56) showed the identical highest efficiency. In comparison to no ethephon application, the energy efficiency of the maize plant increased by 30.71% and 26.77%, respectively, because of a single application of a lower dosage of ethephon at the 6 and 10-leaves stages.

##### Profitability: Gross Return (GR), Gross Margin (GM), and Marginal Benefit–Cost Ratio (MBCR)

The market value of output is treated as gross return (GR), gross return minus production cost is referred as gross margin (GM), and total of the partial budget analysis is known as marginal benefit–cost ratio (MBCR). This is the marginal benefit to marginal cost ratio. The economic analysis for maize productivity per hectare indicated that single application of a lower dosage of ethephon at the 6-leaves stage (T1) provided the highest economic benefit under rainfed conditions (Table 6). The highest GR was obtained from T1 (1446.50 USD ha^−1^) followed by T3 (1405.25 USD ha^−1^) and it was lowest in T0 (1105.50 USD ha^−1^). The GM was maximum in T1 (682.02 USD ha^−1^), which was 53.70% higher than the minimum GM obtained from T0 (443.73 USD ha^−1^). The additional gross returns were 341.00, 255.75, 299.75, 148.50, 60.50, and 134.75 USD ha^−1^, in T1, T2, T3, T4, T5, and T6, respectively, compared to T0. The additional production cost was 102.71, 205.50, 102.71, 180.00, 360.00, and 180.00 USD ha^−1^, in T1, T2, T3, T4, T5, and T6, respectively, compared to T0. However, the maximum marginal benefit–cost ratio (MBCR) was in T1 (3.32), followed by T3 (2.92) and T2 (1.24). The lowest MBCR was in T5 (0.17).

## 3. Discussion

Water availability, like other environmental factors, affects various agro-physio-biochemical traits of maize plants [88,89,90]. In the current study, we compared the agronomic, physiological, and biochemical analyses under water stress conditions in a greenhouse and in a field that was rainfed. The environment and soil factors influence water stress conditions. Crop stress can be mitigated by effective management. Ethephon is a plant growth regulator that improved the morphological, physiological, and biochemical properties of maize plants in the current study, and it was discovered to be a viable technique for improving maize plant performance under rainfed conditions.

Fernandez [91] gave various indices that can be used to rate a genotype’s performance in both control and stress situations. The tolerance index, which included the yield index (YI), identified cultivars with high plant biomass production in both favorable and unfavorable moisture conditions. The results of the selection-based YI were useful in differentiating genotypes in the greenhouse experiment. Suwan 2301, found to be more tolerant and produced plant biomass effectively under both favorable and unfavorable circumstances.

In the case of long-term water stress of the greenhouse study, the PCA biplot PC1 (94.6%) and PC2 (5.4%) results were used to create the first two PCs, which together accounted for 100% of the variance (Figure 3). Kaya et al. [92] discovered that wheat genotypes with higher PC1 and lower PC2 values had greater yields (stable genotypes), while genotypes with lower PC1 and higher PC2 scores had lower yields (unstable genotypes). Under both stressful and non-stressful conditions, genotypes with high PC1 values are anticipated to provide high yields [93]. Golabadi et al. [94] showed durum wheat had similar outcomes. Using principal component analysis, [95] experiments for finding genotypes resistant to drought and susceptible to it revealed two components that accounted for 99% of the variability. Najaphy and Geravandi [96] performed principal component analysis on bread wheat and discovered that selecting high values of YI and YSI and low values of SSI and TOL results in the identification of stress-tolerant and high-yielding genotypes under stress circumstances. A biplot of PC1 and PC2 clearly depicts the correlations between different indices. The PC1 and PC2 axes, which account for 100% of total variation, primarily differentiate the indices into groups. The cosine of the angle between the vectors of two indices approximates the correlation coefficient between them, according to one interesting interpretation of the biplot [97]. Because the biplot does not explain all of the variation in a dataset, the cosines of the angles does not perfectly transfer into correlation coefficients. Nonetheless, the angles are sufficiently informative to provide a comprehensive picture of the interrelationships between the in vivo indices [98].

### 3.1. Climate and Weather Patterns, and Affected Crop Stages in the Field

Rainfall is the primary supply of water for rainfed agriculture, and both its intensity and quantity are impacted by climate change [10,18,19,99]. In Thailand, rainfall uncertainty and temperature fluctuations are increasingly commonplace [11,100,101]. During the crop growth season, the weather at the current experimental location differed slightly from long-term historical data (Figure 5). During the experimental year, the cool season (November to February) became somewhat cooler, while the hot season (March to May) became hotter. The rainfall pattern also diverged from the long-term statistics, with the experimental period having relatively more rainfall than the long-term average. According to [84,85], maize requires about 650 mm of water in total. The amount of rainfall for the current crop cycle was approximately 813 mm, which would have been adequate, but unfortunately, the distribution of the rainfall was not proper. There was almost regular rainfall after maize planting; however, there was only 2.49 mm of rainfall from the 5- to 11-leaves stage of more than 2 weeks, and again during the pre-flowering to flowering stages of about 3 weeks, there was only 7.76 mm rainfall. In contrast, according to [86,87], maize requires approximately 40 mm of water per week for proper plant growth in that location’s reddish-brown lateritic soil. As a result of the lack of rainfall throughout these periods, the crop suffered shortages of water. Moreover, the flowering stage is one of the most critical periods of maize, and water stress in this period decreased maize yield significantly [41,42,43,44,89,102].

### 3.2. Agro-Physio-Biochemical Performance of Maize

The genotypic performance of maize under water stress in the greenhouse revealed that Suwan 2301 (G1) showed better performance in the case of maximum observed traits. Among the drought tolerance indices based on total plant biomass, the yield index (YI) was found more suitable to assess the performance of different traits simultaneously in GH and field levels. Depending on the YI values, different traits were evaluated and selected from GH for triggering them at the field level under rainfed conditions through ethephon application as per the aim of the study. However, the performance of different agronomic, agro-physiological, and physio-biochemical traits of maize under the GH and at field level are discussed below.

### 3.3. Agronomic Performance of Maize under Greenhouse and Field Experiments

In the GH, maize genotype Suwan 2301 produced maximum plant biomass under both short- and long-term water stress, indicating that it may have better stress tolerance capacity against water shortage. However, the tolerance level measured by yield index (YI) showed that the YI values of different agronomic traits, i.e., SBD3, LA3, APB3, RW3, TPB3, and RL3 were higher and PH3 was slightly lower in the best-performer genotype Suwan 2301 in the GH (Figure 4). For this reason, these traits were considered with some additional traits at the field level during the reproductive stage for evaluation under rainfed conditions with a locally popular maize, cultivar Suwan 5819. At the field level, the application of ethephon successfully influenced most of the selected traits. Among the different tactics of ethephon applications, the T1 (ethephon @ 281 g a.i. ha^−1^ applied at the 6-leaves stage of maize) had more triggering capacity for influencing the agronomic traits, i.e., SBD-VT, APB-VT, LA-VT, and additionally KNP, and 100-KW under rainfed conditions compared to no ethephon application. The YI value for plant height (PH-PM) at the field level was likewise somewhat lower in T1, which was also ideal for protecting lodging, and this matched the results of the GH study that showed a lower YI value for plant height in G1. The root length (RL) and root weight (RW) could not be measured at the field level.

According to Jaleel et al. [103], plant responses to water stress differ greatly based on the type of plant and its growth stage [89], as well as the severity and duration of the stress as it coincides with the current GH experiment. Plants exhibit several adaptations to endure the effects of drought, including the development of deep root systems that facilitate better water absorption from the soil’s deep layers, even in dry conditions [12]. Thus, in the current greenhouse investigation, it was discovered that the drought-tolerant genotype’s pot soil had a comparatively lower moisture content and could continue to absorb water even in dry conditions. As noted by Hayano-Kanashiro et al. [104] and confirmed in this study, water scarcity had a substantial impact on the growth [27,29] and development of the aboveground components [105], such as plant height, leaf area, and leaf greenness, during the early vegetative phase. Under water stress, the plant height-lowering rate of different genotypes varied, and these findings are validated by other researchers [41,90,106,107]. Water stress reduced maize plant stem diameter [107,108], although it was less reduced in Suwan 2301, which could be regulated by total dry matter production [41] and water reservation. Furthermore, a deficiency of water has a major impact on the growth and development of roots [41,109]. Suwan 2301, a tolerant genotype, showed the longest roots among the cultivars. In contrast, plant height decreases because of water stress, and it is mainly due to decreases in cell enlargement [110].

Plants that are applied ethephon may initiate ethylene signaling [111] and produce more auxin than cytokinin, which could impact root growth as opposed to shoot growth. For this reason, it is possible that a larger ethephon dosage caused the plant to not grow as tall, and [55,112] supports this theory. Conversely, ethephon may stimulate plants to create more ABA (abscisic acid), which could result in a reduction in plant height [111]. By decreasing stem cell division and elongation, ABA reduces plant height [113]. According to Karimi et al. [114], ethephon treatment at the proper dosage increased stem diameter while slightly lowering plant height. Moreover, an increased stem diameter might help support the plant’s weight, which is essential to avoiding lodging [71]. Even though the maize plant’s height was somewhat reduced, possibly because of the thickening of the stem diameter with a good amount of leaf area, the application of an appropriate dosage (T1) of ethephon preserved the aboveground plant biomass. However, the aboveground plant biomass was decreased by overdosage of ethephon treatment, such as T5 and T2 (Figure 6), which also concurs with Zhang et al. [113]. Water stress causes maize plants to produce fewer grains; however, when applied in the right amount, ethephon can help protect against excessive water loss through transpiration by reducing leaf area and preserving soil water for use later in the reproductive phase [27,57]. The decrease in grain weight may result from the plants receiving less water and nutrients when they are stressed by the water, and ethephon treatment may aid in decreasing water loss and enhance nutrient availability to the kernels by remobilization [59,115]. The application of ethephon at the appropriate dose and plant stage increased maize kernel yield, which could be attributed to reduced water loss and increased nutrient availability to the kernels, increased number of kernels per ear, individual kernel weight, and increased assimilation translocation [55,56,59,113,116].

### 3.4. Agro-Physiological Performance of Maize under Greenhouse and Field Experiments

The YI values of different agro-physiological traits, i.e., RGR3 were higher and NAR-T3, was moderate in the best-performer genotype Suwan 2301 in the GH (Figure 4). To evaluate these characteristics under rainfed conditions, a few more traits were also considered at the field level during the reproductive stage. Most of the chosen features could be successfully influenced by ethephon application at the field level. Out of all the ethephon application strategies, the T1 was more capable of triggering the agro-physiological traits (RGRA-V6R3, NARA-V6R3, and additionally SLW-VT, AGDDs, and HUE) under rainfed conditions than the other tactics. The results showed that T1 had lower RSR-VTR3 and LP-PM, which was also preferable for tolerance.

According to Huo et al. [117], there is evidence that when plants lack water, their ability to capture light is reduced, which in turn leads to a reduction in cellular division and the accumulation of dry matter [26,28]. In the current study, the decrease in dry matter under water stress may have been caused by a decrease in chlorophyll content, which in turn led to a decrease in net assimilation rate and a subsequent reduction in plant growth rate [35,36,37,109,118].

Ethephon application might mitigate this reduction, as indicated by the current experiment. This might be due to a strong root system supported by a thicker stem, higher relative water content to transport sufficient nutrients, and acceptable assimilate production through a satisfactory green leaf area [56,113]. The current study showed that ethephon application reduced leaf area but increased specific leaf weight, which was also found by several investigators [56,119] who reported that thicker leaves (greater SLW) increased stomatal density, enhanced chlorophyll and protein contents, increased CO_2_ exchange rate, and yield of maize. Growing conditions, particularly at changing water levels, might cause variations in accumulated GDDs [120,121]. Overdosages of ethephon application decreased maize GDDs by causing early plant senescence. This is because ethephon might cause the plant to stop growing and, at the end of the reproductive phase, begin to die sooner than it would under normal conditions. However, at the optimum ethephon dosage, it was balanced by balancing root growth, leaf area, and leaf greenness. Depending on the growing conditions, maize’s heat consumption efficiency may vary [122]. Due to a lack of water, maize HUE decreased, which could be related to the plant’s ability to absorb water and nutrients. As a result, plant growth and development may be slowed, resulting in decreased biomass production. The application of ethephon altered this situation by stimulating the stress-tolerant characteristics. In the current experiment, however, heavy rainfall with wind during the later growth stage of maize induced maize plant lodging, which was greatly reduced in the ethephon-treated plot. Plant lodging can impact photosynthesis, pollination, kernel setting, kernel filling, and yield of maize due to poor solar radiation interception and nutrient transportation [54,55,69,123,124,125]. However, the application of ethephon can significantly lower the percentage of lodged plants [55,71,72,123,124,125,126].

### 3.5. Physio-Biochemical Performance of Maize under Greenhouse and Field Experiments

The best-performing genotype, Suwan 2301, had higher YI values for several physio-biochemical characteristics, such as RWC3, SPAD3, Pr3, and TSS3 in the GH (Figure 4). A few other attributes were also considered at the field level to evaluate these characteristics under rainfed conditions. At the field level, ethephon application could successfully influence most of the selected features. Compared to the other ethephon application procedures, the T1 was better at triggering the physio-biochemical characteristics (RWC-VT, SPAD-VT, Pr-VT, TSS-VT, and additional EL-VT and Ca) in rainfed conditions. According to the data, T1 had lower EL-VT, which was also a desirable characteristic for tolerance.

Drought causes a decrease in plant water content, according to various studies [127,128,129,130,131,132,133], where under well-watered condition leaf water content may not differ between tolerant and susceptible genotypes [134]. The current study’s findings demonstrated that relative water content reduced as the water stress period increased. Water scarcity had a substantial impact on the leaf greenness during the early vegetative phase [27,29,109] and this result coincides with the current study. Under long-term water stress, the proline and total soluble sugar content were increased, and it was prominent in G1. In accordance with several studies, proline, total soluble sugar, relative water levels, and other factors are crucial for determining how well maize withstands water stress [45,46,47,48,49].

Insufficient soil water decreased the relative water content (RWC) of maize under water stress circumstances [41,109], whereas ethephon treatment preserved the RWC of maize leaves under water shortage conditions [135]. The reason for this could be that the ethephon promotes stomata to close, which lowers water loss from maize leaves [62]. Ethephon application influenced the production of more proline in maize plants under rainfed conditions, and this result was also supported by [59,62,135,136]. Under water stress circumstances, total soluble sugar can operate as an osmoregulatory solute. A range of organic compatible solutes given either before or during environmental stress may protect plants from damage by boosting internal plant solute levels [64,65,66,67]. The current study discovered that T1 generated higher TSS, which may protect the maize plant under rainfed conditions from potential yield losses [137]. This might be because ethephon helps break down starch [111] into sugars that are soluble. Applying ethephon reduced the electrolyte leakage of maize leaves during drought stress [74]. Ethephon can improve cell membrane integrity by promoting the synthesis of specific proteins and enzymes that aid in shielding the membranes from harm [135]. Conversely, reactive oxygen species (ROS) generated under water stress have the potential to harm cell membranes and cause electrolyte leakage [138]. Through boosting antioxidant production, ethephon helps lessen oxidative stress in maize plants [59]. Additionally, ethephon supports the growth of the root system, which might help the plant absorb and hold onto more water, thus protecting the EL.

### 3.6. Sustainability of Maize Production Using Ethephon in Rainfed Conditions

An agricultural production system’s sustainability can be evaluated using a number of different indicators. According to the findings of Haarhoff et al. [82], economic analysis of a production system, productivity of crops, and resource usage efficiency may all be useful indicators of sustainability. Sustainable maize production in rainfed areas may be characterized by increased profitability and productivity with environmentally friendly tactics.

Among the different tactics of ethephon applications, the T1 (ethephon @ 281 g a.i. ha^−1^ applied at the 6-leaves stage of maize) was more efficient for increasing crop productivity, heat use efficiency, energy efficiency, carbon absorption, and profitability. According to the current study, excessive ethephon application doses decrease AGDDs, which lowers HUE. Furthermore, the HUE could be raised and the AGDDs could be maintained with the proper amount of ethephon as the T1 application. These findings corroborated the findings of Singh and Hadda [122], who observed that lower AGDDs lowered HUE and maize grain production.

Rising CO_2_ concentrations in the atmosphere are widely acknowledged as the major cause of global climate change [139]. The current study’s results demonstrate that ethephon treatment has a considerable effect on maize C absorption. In comparison to no ethephon treatment (T0), ethephon application as T1 enhanced C absorption by 20% (Figure 10A). Several studies [139,140,141] found that high C absorption enhances both above- and belowground biomass in maize, resulting in increased yield.

The environmental effect of producing maize can be reduced with the use of energy use efficiency [142]. One major input cost that maize growers face is energy. By raising energy use efficiency, farmers may reduce production costs and raise profitability. When applied at the V6 stage of maize at 281 g a.i. ha^−1^ (T1), ethephon can increase energy use efficiency under rainfed conditions by decreasing the need for water through increased soil water absorption, enhancing fertilizer use efficiency through increased nutrient uptake and utilization [113], and enhancing photosynthesis efficiency through increased chlorophyll production [135]. However, ethephon application can lead to improved maize output with lower energy inputs by improving water stress tolerance, boosting root growth, reducing lodging [113,125,143], more N allocation to grains rather than stover [113], lowering water loss and electrolyte leakage [135,138], improving photosynthesis [113], and reducing stress [55,111].

Crop production profitability is always a concern for the farming community [144]. Based on kernel yield from ethephon application compared to no ethephon plots, an economic evaluation for profitability was carried out. This study showed that applying ethephon at a lower dosage was profitable when it came to kernel yield. The ethephon application with T1 received the highest ratings for gross margin and benefit–cost ratio; another option would be the ethephon application with T3. Consequently, applying ethephon to maize in the six-leaves stage at a rate of 281 g a.i. ha^−1^ may be more appropriate.

## 4. Materials and Methods

The field experiment was conducted in 2020 at Pakchong, Nakhon Ratchasima, Thailand, and was preceded by a greenhouse (GH) experiment in 2019 at Kasetsart University, Thailand.

### 4.1. Design with Treatments, Plant Materials, Cultural Management, Soil, Climate, and Weather

#### 4.1.1. Greenhouse Experiment

Design, treatments, and plant materials: The GH experiment was laid out in a randomized complete block (RCB) design maintaining factorial arrangement with two factors in four replications. The first factor was three maize genotypes (G), i.e., Suwan 2301 (G1), Suwan 4452 (G2), and S 7328 (G3), where three water (W) levels were the second factor, i.e., watering every day (W1) as well watered, watering every 2 days (W2) as short-term water stress, and watering every 4 days (W3) as long-term water stress. The maize genotypes were chosen for the greenhouse experiment by guessing their drought tolerance capacity (G1 > G2 > G3) as per Kanavittaya et al. [145].

Cultural management, soil and weather: The maize seeds were disinfected with a 2% sodium hypochlorite (NaOCl) solution and then imbibed in distilled water for 24 h. Disinfected maize seeds were sown on 17 November 2019 in a five-liter size pot filled with four kilograms of air-dried mixed soil (1:1 ratio of Chia Tai soil and Field soil). The soil was collected from a maize growing area in Pakchong, Nakhon Ratchasima, Thailand, which had a silty clay texture with a sand content of 9.61%, silt content of 40.22%, and clay content of 50.21%. Its characteristics included nitrogen of 0.09%, phosphorus of 72 mg kg^−1^, potassium of 140 mg kg^−1^, and organic matter of 2.29%. In terms of treatment, deficit watering was started 16 days after planting (DAP) and at each watering time, 1750 mL water was supplied in the morning. Prior to that, all the pots had adequate soil moisture for easy germination and early plant growth. A WET-2 sensor (Delta-T Devices, Cambridge, UK) was used throughout the experiment to measure the volumetric soil moisture content every 12 h (at 7:00 a.m., just before watering, and 7:00 p.m.). Under the tropical environment, the daily maximum, minimum, and average temperature ranges were 30.0–32.8 °C, 22.2–27.2 °C, and 26.9–30.1 °C, respectively, whereas the relative humidity ranges were 74–100%, 38–62%, and 59.6–82.5%, respectively, during the experimental period.

#### 4.1.2. Field Experiment

Design, treatments, and plant materials: The field experiment was carried out using an RCB design maintaining four replications. Six ethephon (E) application tactics (dosage in g a.i. ha^−1^), i.e., ethephon @ 281 at V6 stage (T1), ethephon @ 281 at V6 + 281 at V10 stage (T2), ethephon @ 281 at V10 stage (T3), ethephon @ 562 at V6 stage (T4), ethephon @ 562 at V6 + 562 at V10 stage (T5), and ethephon @ 562 at V10 stage (T6) were tested against no ethephon application (T0). The maize cultivar Suwan 5819 was chosen based on the local popularity (farmers’ choice).

Cultural management: Seeds of maize were planted in the field during the rainy season on 5 July 2020, maintaining a spacing of 70 cm × 20 cm. The fertilizer NPK (16:16:16) @ 313 kg ha^−1^ was applied as basal, and 46 N @ 313 kg ha^−1^ was top-dressed at 22 days after planting (DAP). No irrigation was provided during the maize growing season in the field.

*Soil:* The characteristics of the surface soil (0–30 cm) were as follows: organic matter (OM) content: 2.27% (moderate), total N: 0.12% (low), available P: 73 mg kg^−1^ (very high), exchangeable K: 41 mg kg^−1^ (low), pH: 6.8 (neutral), sand: 15%, silt: 10%, and clay: 75% (clay soil).

**Figure 11:**
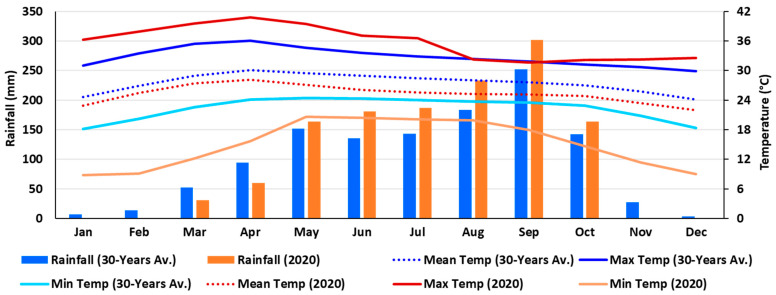
Monthly rainfall (total) and temperature (maximum, mean and minimum) for long-term of 30 years average (1991–2020) as well as the present experimental year (2020) at the trial location, Pakchong, Nakhon Ratchasima, Thailand.

Climate and weather: Long-term data on rainfall and temperature (maximum, minimum, and average) of the trial location were retrieved from World Bank sources for the yearly average data from 1901 to 2021 (Figure S1) [145], and monthly average data from 1991 to 2020 (Figure 11). The weather data for the experimental year (2020) were collected from the local weather station near the experimental plot (Figure 11) [100].

### 4.2. Measurements of Different Traits and Plant Sampling in Greenhouse and Field Experiments

Different data on agro-physio-biochemical traits of maize were collected from both greenhouse (GH) and field experiments by three measurements of each parameter. In the case of the GH experiment, data were collected at 5, 9, and 13 days after the watering treatment started (DAWTS), and the watering treatments were started 16 days after planting (DAP). Data from the field experiment were collected at the vegetative 6-leaves stage (V6), vegetative 10-leaves stage (V10), vegetative tasseling stage (VT), reproductive phase 3 of maize plant (R3), physiological maturity stage (PM), and final harvest (FH).

The relative growth rate (*RGR*) was calculated as follows according to Farahani et al. [146]:

$$RGR\;\left({\mathrm{mg}\;\mathrm{plant}}^{-1}{\;\mathrm{day}}^{-1}\right)=\left(Ln\;W_{2}-Ln\;W_{1}\right)/\left(T_{2}-T_{1}\right)$$

*W*_1_: plant dry weight at time *T*_1_; *W*_2_: plant dry weight at time *T*_2_; *T*_1_ and *T*_2_: time interval in days; and *Ln*: natural logarithm.

The leaf area index (*LAI*) was computed using the following formula according to Molla et al. [147], where the leaf area is the one-sided green leaf area per unit ground surface area:

LAI=Leaf area/Ground area

Plant-wise leaf area (*LA*) was measured according to Molla et al. [109]:

$$LA\;\left({\mathrm{cm}}^{2}{\;\mathrm{plant}}^{-1}\right)=Length\times Maximum\;width\times 0.75$$

Specific leaf weight (*SLW*) was measured at tasseling stage from ear leaves according to Farahani et al. [146]:

$$SLW\;\left({g\;m}^{-2}\right)=Leaf\;dry\;weight\;\left(g\right)÷Leafarea\;\left(m^{2}\right)$$

The net assimilation rate (*NAR*) was estimated according to Diaz-Lopez et al. [148]:

$$NAR\;\left({\mathrm{mg}\;\mathrm{cm}}^{-2}{\;\mathrm{day}}^{-1}\right)=\left(\frac{W_{2}-W_{1}}{T_{2}-T_{1}}\right)\times \left(\frac{Ln\;LA_{2}-Ln\;LA_{1}}{LA_{2}-LA_{1}}\right)$$

where *W*_2_ and *W*_1_ are the dry weights of the plant at times *T*_2_ and *T*_1_, where *LA*_2_ and *LA*_1_ are the leaf areas at times *T*_2_ and *T*_1_, respectively.

The relative water content (*RWC*) of ear leaf was estimated according to Molla et al. [109]:

$$RWC\;\left(\%\right)=\left(Fresh\;weight-Dry\;weight\right)\times 100/\left(Turgid\;weight-Dry\;weight\right)$$

The relative senescence rate (*RSR*) was estimated from green leaf number at VT to R3 stage according to Molla et al. [105]:

$$RSR\;\left(\%\right)=\frac{\left(GLN\;t_{1}-GLN\;t_{2}\right)}{\left(t_{2}-t_{1}\right)}\times \frac{2}{\left(GLN\;t_{1}+GLN\;t_{2}\right)}$$

where *GLN*: green leaf number (≥50% green), *t*_1_: days to *VT* stage, *t*_2_: days to R3 stage.

The electrolyte leakage (*EL*) was measured from leaf discs following the methods of Dionisio-Sese et al. [149]:

$$EL\;\left(\%\right)=\frac{EC1}{EC2}\times 100$$

The total soluble sugar (*TSS*) content was measured from leaves following the methods described by Irigoyen et al. [150]. A spectrophotometer set at 620 nm was used to measure the total soluble sugar content using a standard curve.

The proline (pr) content in leaves was measured following the methods of Bates et al. [151]. A spectrophotometer was set at 520 nm to measure the pr and pure proline was used to construct a standard curve.

Twenty drought tolerance indices were calculated based on total dry biomass under short-term water stress and long-term water stress conditions in the greenhouse to identify the suitable index system for maize. Among them, the yield index (YI), one of the suitable indices, was further used to calculate the indices based on different agro-physio-biochemical traits of maize in the greenhouse (GH) as well as at the field level (FL). The equations listed in Table 7 were used to calculate drought tolerance indices.

The accumulated growing degree days (*AGDDs*) were estimated as per the following formula according to Singh and Hadda [122]:

$$AGDD\;\left({}^{\circ}C\;\mathrm{days}\right)=\overset{n}{\underset{i=1}{\sum }}T_{i}-T_{b}$$

$$HUE\;\left({\mathrm{kg}\;\mathrm{ha}}^{-1}\;{}^{\circ}{C\;\mathrm{days}}^{-1}\right)=Grain\;yield\;in\;{\mathrm{kg}\;\mathrm{ha}}^{-1}/AGDD\;in\;{}^{\circ}C\;\mathrm{days}$$

where *i* denotes the *i*th day after sowing, Ti denotes the average temperature for that day, *n* denotes the number of days in the growing season, and *T_b_* denotes the base temperature (10 °C).

The heat use efficiency (*HUE*) was estimated as follows according to Singh and Hadda [122]:

$$HUE\;\left({\mathrm{kg}\;\mathrm{ha}}^{-1}\;{}^{\circ}{C\;\mathrm{days}}^{-1}\right)=Grain\;yield\;in\;kg\;ha^{-1}/AGDD\;in\;{}^{\circ}C\;days$$

According to Feng et al. [139] and Li [166], the *C* absorption (*C_a_*) rate at the maturity stage of maize was estimated as follows:

Cat C ha−1=Cf×Dw=Cf×YwHi

where *C_a_* represents the carbon (*C*) absorption by maize at the maturity stage and *C_f_* represents the effectiveness of the *C* source in the synthesis of dry matter (0.4709). The economic yield is *Y_w_*. *D_w_* is the organic yield, while *H_i_* is the index (0.40).

Energy use efficiency (EUE) was calculated according to Laskari et al. [106] using the energy equivalents of the inputs and outputs (Table 8). The conversion of all production components utilized in the maize production system and products into energy units was used for the energy approach.

Profitability: The profitability of a production system can be assessed by the cost and return analysis included gross return (GR), gross margin (GM) and marginal benefit–cost ratio (*MBCR*). The inputs and output were valued at existing market prices and converted to USD. The MBCR of the control plot and any replacement for it can be computed as the marginal value product (*MVP*) over the marginal value cost (*MVC*). The marginal value of the control plot (*C*) and any potential replacement (*E*) for it was computed as per Chowdhury et al. [172] and CIMMYT [173]:

$$Marginal\;benefit\;cost\;ratio\;\left(MBCR\right)=\frac{Gross\;return\;\left(E\right)-Gross\;return\;\left(C\right)}{TVC\;\left(E\right)-TVC\;\left(C\right)}=\frac{MVP}{MVC}$$

where *MVP*: marginal value of product, *MVC*: marginal value of cost; calculated over control (*C*), i.e., no ethephon treatment (T0); gross return: the market value of output; *TVC*: total variable cost.

### 4.3. Data Analysis

Analysis of variance (ANOVA) was used for various data in a factorial randomized complete block (RCB) design for the greenhouse experiment and RCB design for the field experiment. Means and standard deviations are used to present the trial’s findings. At *p* = 0.05, Fisher’s protected least significant difference (LSD) approach was used to compare the mean values in appropriate cases. The analysis was conducted using the statistical program SPSS for Windows, version 16.0. Chicago, IL, USA, SPSS Inc., MS Excel, CropStat 7.2 (International Rice Research Institute, Los Baños, Philippines), R [174] version 4.2.3, and RStudio statistical packages together with the ggplot2 package. A principal component analysis (PCA) biplot was produced using the two R packages factoextra and FactoMineR. This PCA only employs the two treatments of short-term water stress and long-term water stress for analysis to reflect the effects of water stress more accurately on crop growth in the greenhouse. In order to further illustrate the effects of ethephon treatment on rainfed maize at the field level, path analysis was performed using data on suitable variables treated with ethephon levels in three separate groups (agronomic, agro-physiological, and physio-biochemical).

## 5. Conclusions

Climate change affected the weather pattern remarkably, and it was found that in the tropical country of Thailand, the hot season is becoming hotter, rainfall is irregular, heavy rainfall occurs during the late rainy season to the early dry season, and the dry season is extending. Under these circumstances, the present experiment showed that maize production with ethephon as T1 tactics (ethephon application @ 281 g a.i. ha^−1^ at the 6-leaves stage of maize) may reduce yield loss as well as increase profitability and sustainability under irregular rainfall in the rainy season. It was revealed that T1 tactics influenced the agro-physio-biochemical traits of maize at the field level under rainfed conditions remarkably, which was ascertained in the greenhouse experiment. However, from the GH experiment, it was revealed that Suwan 2301 (G1) is more tolerant to water stress compared to other genotypes. This genotype showed better performance in SBD, RWC, Pr, TSS, APB, NAR, and RGR, even under long-term water stress. These traits also showed more tolerance against water stress in terms of yield index (YI). The target of our study was identifying the tolerance traits at GH. Efforts would be made at field level to enhance these through ethephon application under rainfed conditions. The findings of the current study showed that the ascertained characteristics could be triggered successfully by application of ethephon, especially application of T1 tactics. However, this dosage at the 10-leaves stage (T3) may be an alternative option, as it performed almost the same as T1. Regarding sustainability, the T1 tactics were also more effective, resulting in more productivity, energy efficiency, heat use efficiency, carbon absorption, and profitability. Nonetheless, caution needs to be taken against excess dosage. Further research is needed on molecular analysis in different countries of the tropics to enhance the sustainability of maize production under rainfed as well as water deficit conditions.

## Figures and Tables

**Table 1:** The drought tolerance indices and total plant biomass per plant of three maize genotypes considering well-watered and long-term water-stressed conditions.

	Yp	Ys	MP	MRP	SSI	TOL	GMP	REI	STI	MSTIk1	MSTIk2	HARM	YI	Red	RDI	DI	GM	ATI	SSPI	SNPI	RDY
G1	20.16	12.72	16.44	2.19	0.84	7.44	16.02	1.19	0.69	0.70	0.90	15.60	1.16	36.90	1.13	0.73	4.42	66.53	18.96	4.03	97.43
G2	20.31	11.70	16.00	2.10	0.96	8.61	15.41	1.10	0.62	0.66	0.70	14.85	1.07	42.41	1.03	0.61	3.72	71.12	21.94	3.74	97.62
G3	18.40	8.45	13.43	1.71	1.22	9.95	12.47	0.72	0.40	0.35	0.24	11.58	0.77	54.08	0.82	0.35	2.70	69.27	25.35	3.09	98.45

G1, Suwan 2301; G2, Suwan 4452; G3, S 7328; MP, mean productivity; MRP, mean relative performance; SSI, stress susceptibility index; TOL, tolerance index; GMP, geometric mean productivity; REI, relative efficiency index; STI, stress tolerance index; MSTIk1, modified stress tolerance index 1; MSTIk2, modified stress tolerance index 2; HARM, harmonic mean of yield; YI, yield index; Red, reduction; RDI, relative drought index; DI, drought resistance index; GM, golden mean; ATI, abiotic tolerance index; SSPI, stress susceptibility percentage index; SNPI, stress/non-stress production index; RDY, relative decrease in yield.

**Table 2:** Correlation coefficients of total plant biomass grown under well-watered conditions (Yp), total plant biomass grown under stressed conditions (Ys), and several drought tolerance indices of three maize genotypes.

	Yp	Ys	MP	MRP	SSI	TOL	GMP	REI	STI	MSTIk1	MSTIk2	HARM	YI	Red	RDI	DI	GM	ATI	SSPI	SNPI	RDY
Ys	0.95 *																				
MP	0.98 *	1.00 *																			
MRP	0.97 *	1.00 *	1.00 *																		
SSI	−0.93 *	−1.00 *	−0.98 *	−0.99 *																	
TOL	−0.85 *	−0.97 *	−0.94 *	−0.95 *	0.99 *																
GMP	0.97 *	1.00 *	1.00 *	1.00 *	−0.99 *	−0.95 *															
REI	0.97 *	1.00 *	1.00 *	1.00 *	−0.99 *	−0.95 *	1.00 *														
STI	0.95 *	1.00 *	1.00 *	1.00 *	−1.00 *	−0.97 *	1.00 *	1.00 *													
MSTIk1	0.98 *	0.99 *	1.00 *	1.00 *	−0.98 *	−0.93 *	1.00 *	1.00 *	0.99 *												
MSTIk2	0.93 *	1.00 *	0.99 *	0.99 *	−1.00 *	−0.98 *	0.99 *	0.99 *	1.00 *	0.98 *											
HARM	0.97 *	1.00 *	1.00 *	1.00 *	−0.99 *	−0.95 *	1.00 *	1.00 *	1.00 *	1.00 *	0.99 *										
YI	0.96 *	1.00 *	1.00 *	1.00 *	−1.00 *	−0.97 *	1.00 *	1.00 *	1.00 *	0.99 *	1.00 *	1.00 *									
Red	−0.92 *	−1.00 *	−0.98 *	−0.99 *	1.00 *	0.99 *	−0.99 *	−0.99 *	−1.00 *	−0.98 *	−1.00 *	−0.99 *	−1.00 *								
RDI	0.92 *	1.00 *	0.98 *	0.99 *	−1.00 *	−0.99 *	0.99 *	0.99 *	1.00 *	0.98 *	1.00 *	0.99 *	1.00 *	−1.00 *							
DI	0.93 *	1.00 *	0.98 *	0.99 *	−1.00 *	−0.99 *	0.99 *	0.99 *	1.00 *	0.98 *	1.00 *	0.99 *	1.00 *	−1.00 *	1.00 *						
GM	0.88 *	0.98 *	0.96 *	0.97 *	−0.99 *	−1.00 *	0.97 *	0.97 *	0.98 *	0.95 *	0.99 *	0.97 *	0.98 *	−1.00 *	1.00 *	0.99 *					
ATI	−0.04 ^ns^	−0.34 ^ns^	−0.24 ^ns^	−0.28 ^ns^	0.41 ^ns^	0.56 *	−0.27 ^ns^	−0.29 ^ns^	−0.34 ^ns^	−0.21 ^ns^	−0.40 ^ns^	−0.28 ^ns^	−0.33 ^ns^	0.42 ^ns^	−0.42 ^ns^	−0.41 ^ns^	−0.50 *				
SSPI	−0.85 *	−0.97 *	−0.94 *	−0.95 *	0.99 *	1.00 *	−0.95 *	−0.95 *	−0.97 *	−0.93 *	−0.98 *	−0.95 *	−0.97 *	0.99 *	−0.99 *	−0.99 *	−1.00 *	0.56 *			
SNPI	0.93 *	1.00 *	0.99 *	0.99 *	−1.00 *	−0.98 *	0.99 *	0.99 *	1.00 *	0.98 *	1.00 *	0.99 *	1.00 *	−1.00 *	1.00 *	1.00 *	0.99 *	−0.41 ^ns^	−0.98 *		
RDY	−0.97 *	−1.00 *	−1.00 *	−1.00 *	0.99 *	0.95 *	−1.00 *	−1.00 *	−1.00 *	−1.00 *	−0.99 *	−1.00 *	−1.00 *	0.99 *	−0.99 *	−0.99 *	−0.97 *	0.28 ^ns^	0.95 *	−0.99 *	
DTE	0.92 *	1.00 *	0.98 *	0.99 *	−1.00 *	−0.99 *	0.99 *	0.99 *	1.00 *	0.98 *	1.00 *	0.99 *	1.00 *	−1.00 *	1.00 *	1.00 *	1.00 *	−0.42 ^ns^	−0.99 *	1.00 *	−0.99 *

MP, mean productivity; MRP, mean relative performance; SSI, stress susceptibility index; TOL, tolerance index; GMP, geometric mean productivity; REI, relative efficiency index; STI, stress tolerance index; MSTIk1, modified stress tolerance index 1; MSTIk2, modified stress tolerance index 2; HARM, harmonic mean of yield; YI, yield index; Red, reduction; RDI, relative drought index; DI, drought resistance index; GM, golden mean; ATI, abiotic tolerance index; SSPI, stress susceptibility percentage index; SNPI, stress/non-stress production index; RDY, relative decrease in yield; DTE, drought tolerance efficiency. * significant at *p* < 0.05; ns: nonsignificant.

**Table 3:** Effect of ethephon application tactics on different agronomic traits of maize under rainfed conditions at field level.

Treatment	PH-PM (cm)	EH-PM (cm)	EH:PH Ratio (%)	SBD-VT (mm)	LA-V6 (cm^2^ Plant^−1^)	LA-V10 (cm^2^ Plant^−1^)	LA-VT (cm^2^ Plant^−1^)	LA-R3 (cm^2^ Plant^−1^)	APB-R3 (g Plant^−1^)	KNP (No. Plant^−1^)	100-KW (g)	KY (t ha^−1^)
T1	192.90 abc	73.58 b	38.15 b	24.49	1247.15	3203.74 b	6107.50 b	4270.00 b	271.34 a	352.25	25.31 a	5.26 a
T2	178.35 c	65.13 c	36.52 bc	24.13	1298.87	3272.50 b	5687.50 bc	3885.00 c	247.14 bc	348.12	24.57 a	4.95 a
T3	193.50 ab	73.01 b	37.73 b	24.42	1278.00	3632.48 a	6020.00 b	4532.50 b	270.35 ab	351.14	25.15 a	5.11 a
T4	183.65 bc	66.45 c	36.18 bc	23.95	1246.52	2697.77 c	5372.50 c	3797.50 c	245.12 c	352.11	24.95 a	4.56 b
T5	160.42 d	55.20 d	34.41 c	23.53	1262.96	2724.45 c	4637.50 d	3412.50 d	235.21 c	340.87	24.12 a	4.24 bc
T6	185.50 bc	65.25 c	35.18 bc	23.90	1269.92	3628.98 a	5425.00 c	3832.50 c	244.68 c	350.14	24.92 a	4.51 b
T0	205.14 a	99.87 a	48.68 a	23.66	1260.00	3650.22 a	6825.00 a	5075.00 a	251.12 abc	334.13	21.56 b	4.02 c
LSD_0.05_	14.57	5.64	2.98	ns	ns	254.99	448.31	324.38	23.66	ns	1.91	0.37

V6: vegetative 6-leaves stage; V10: vegetative 10-leaves stage; VT: vegetative tasseling stage; R3: reproductive phase 3 of maize plant; PM: physiological maturity stage; PH-PM: plant height at PM; EH-PM: ear height at PM; EH:PH: ear height and plant height ratio; SBD-VT: stem base diameter at VT; LA-V6, LA-V10, LA-VT, and LA-R3: leaf area at V6, V10, VT, and R3, respectively; APB-V6, APB-V10, APB-VT, and APB-R3: aboveground plant biomass at V6, V10, VT, and R3, respectively; KNP: kernel number per plant; 100-KW: 100-kernel weight; KY: kernel yield. T1: ethephon @ 281 at V6 stage; T2: ethephon @ 281 at V6 + 281 at V10 stage; T3: ethephon @ 281 at V10 stage; T4: ethephon @ 562 at V6 stage; T5: ethephon @ 562 at V6 + 562 at V10 stage; T6: ethephon @ 562 at V10 stage; T0: no ethephon application; means within a column with the same or no letters are not significant at *p* < 0.05 based on the LSD test.

**Table 4:** Effect of ethephon application tactics on different agro-physio-biochemical traits of maize under rainfed conditions at field level.

Treatment	SLW-VT (g m^−2^)	LP-PM (%)	RGRA-V6R3 (mg Plant^−1^ Day^−1^)	NAR-V6R3 (mg cm^−2^ Day^−1^)	RWC-VT (%)	SPAD-VT	RSR-VTR3 (%)	EL-VT (%)	TSS-VT (mg g^−1^ FW)	Pr-VT (µmol g^−1^ FW)
T1	51.71 a	3.02 c	25.26 a	0.91 a	90.38 ab	42.75	1.67 d	4.91 b	1629.58 a	24.79 a
T2	50.87 a	2.11 d	24.26 b	0.85 ab	89.74 ab	41.02	1.83 abc	4.80 b	1594.23 a	23.98 a
T3	51.27 a	3.80 b	25.16 a	0.88 ab	93.12 a	42.96	1.71 cd	4.95 b	1627.78 a	24.75 a
T4	51.04 a	3.07 c	24.35 b	0.87 ab	90.11 ab	41.32	1.87 ab	5.11 b	1610.12 a	24.15 a
T5	50.64 a	1.89 d	23.70 c	0.82 b	88.12 ab	40.10	1.96 a	5.06 b	1590.21 a	23.90 a
T6	50.79 a	3.75 b	24.30 b	0.84 b	90.04 ab	41.28	1.76 bcd	5.01 b	1605.84 a	24.03 a
T0	40.07 b	14.86 a	24.28 b	0.73 c	85.14 b	40.12	1.63 d	5.90 a	1287.05 b	16.07 b
LSD_0.05_	3.89	0.48	0.14	0.07	7.01	ns	0.14	0.40	122.98	1.83

V6: vegetative 6-leaves stage; V10: vegetative 10-leaves stage; VT: vegetative tasseling stage; R3: reproductive phase 3 of maize plant; PM: physiological maturity stage; SLW-VT: specific leaf weight at VT; LP-PM: lodging percentage at PM; RGRA-V6V10, RGRA-V10VT, and RGRA-V6R3: relative growth rate based on aboveground plant biomass during V6 to V10, V10 to VT, and V6 to R3, respectively; NARA-V6V10, NARA-V10VT, and NARA-V6R3: net assimilation rate based on aboveground plant biomass during V6 to V10, V10 to VT, and V6 to R3, respectively; AGDDs: accumulated growing degree days; HUE: heat use efficiency; RWC-VT: relative water content at VT; SPAD-VT: SPAD value of leaf greenness at VT; RSR-VTR3: relative senescence rate during VT to R3; EL-VT: electrolyte leakage at VT; C: carbon; TSS-VT: total soluble sugar at VT; Pr-VT: proline content at VT. T1: ethephon @ 281 at V6 stage; T2: ethephon @ 281 at V6 + 281 at V10 stage; T3: ethephon @ 281 at V10 stage; T4: ethephon @ 562 at V6 stage; T5: ethephon @ 562 at V6 + 562 at V10 stage; T6: ethephon @ 562 at V10 stage; T0: no ethephon application; means within a column with the same or no letters are not significant at *p* < 0.05 based on the LSD test.

**Table 5:** Indirect effect via various paths of agronomic, agro-physiological, and physio-biochemical traits separately on kernel yield and their correlations under rainfed conditions at the field level.

**Indirect Effect via Agronomic Traits**						
**Trait**	**PH-PM**	**SBD-VT**	**LA-VT**	**100-KW**	**KNP**	**Total Correlation with KY**
PH-PM		0.22	0.85	−0.22	0.01	0.11
SBD-VT	−0.28		0.30	0.41	−0.06	0.96
LA-VT	−0.71	0.19		−0.31	0.02	0.10
100-KW	0.26	0.38	−0.44		−0.08	0.76
KNP	0.06	0.44	−0.22	0.60		0.80
**Indirect effect via agro-physiological traits**						
**Trait**	**SLW-VT**	**LP-PM**	**NARA-V6R3**	**RGRA-V6R3**	**HUE**	**Total correlation with KY**
SLW-VT		0.71	0.19	0.04	0.76	0.67
LP-PM	1.00		−0.17	−0.01	−0.67	−0.57
NAR-V6R3	−0.92	0.59		0.09	0.90	0.87
RGR-V6R3	−0.24	0.04	0.13		0.71	0.79
HUE	−0.80	0.49	0.19	0.11		0.98
**Indirect effect via physio-biochemical traits**						
**Trait**	**RWC-VT**	**SPAD-VT**	**EL-VT**	**TSS-VT**	**Pr-VT**	**Total correlation with KY**
RWC-VT		0.81	0.95	−1.41	0.78	0.81
SPAD-VT	−0.28		0.64	−0.97	0.54	0.89
EL-VT	0.26	−0.52		1.59	−0.89	−0.75
TSS-VT	−0.28	0.56	1.14		0.93	0.68
Pr-VT	−0.27	0.56	1.14	−1.67		0.69

V6, vegetative 6-leaves stage; VT, vegetative tasseling stage; R3, reproductive phase 3 of maize plant; PM, physiological maturity stage; KNP, kernel number per plant; 100-KW, 100-kernel weight; LA-VT, leaf area at VT; SBD-VT, stem base diameter at VT; PH-PM, plant height at PM; SLW-VT, specific leaf weight at VT; LP-PM, lodging percentage at PM; NARA-V6R3, net assimilation rate based on aboveground plant biomass during V6 to R3; RGRA-V6R3, relative growth rate based on aboveground plant biomass during V6 to R3; HUE, heat use efficiency; RWC-VT, relative water content at VT; SPAD-VT, SPAD value of leaf greenness at VT; EL-VT, electrolyte leakage at VT; TSS-VT, total soluble sugar at VT; Pr-VT, proline content at VT.

**Table 6:** Cost-and-return analysis of maize as affected by the magnitude of ethephon application under rainfed conditions at field level.

Ethephon	Kernel Yield (t ha^−1^)	Gross Return (USD ha^−1^)	Total Variable Cost (USD ha^−1^)	Gross Margin (USD ha^−1^)	MBCR (Over No Ethephon T0)
T1	5.26	1446.50	764.48	682.02	3.32
T2	4.95	1361.25	867.27	493.98	1.24
T3	5.11	1405.25	764.48	640.77	2.92
T4	4.56	1254.00	841.77	412.23	0.83
T5	4.24	1166.00	1021.77	144.23	0.17
T6	4.51	1240.25	841.77	398.48	0.75
T0	4.02	1105.50	661.77	443.73	-

T1 = 281 g a.i. ha^−1^ @V6 stage, T2 = 281 g a.i. ha^−1^ at V6 + 281 g a.i. ha^−1^ at V10 stage, T3 = 281 g a.i. ha^−1^ at V10 stage, T4 = 562 g a.i. ha^−1^ @V6 stage, T5 = 562 g a.i. ha^−1^ at V6 + 562 g a.i. ha^−1^ at V10 stage, T6 = 562 g a.i. ha^−1^ at V10 stage, T0 = No ethephon; MBCR = marginal benefit–cost ratio; USD = US dollar; 1 USD≈34 Baht.

**Table 7:** Using the following equations, drought tolerance indices were computed.

Sl.	Index	Equation	Reference	Sl.	Index	Equation	Reference
1.	MP	$\left(Y_{pi}+Y_{si}\right)/2$	[152]	11.	YI	$\left(Y_{si}/Y_{s}\right)$	[153,154]
2.	MRP	YsiYs+YpiYp	[155]	12.	Red	Ypi−YsiYpi×100	[156]
3.	SSI	1−YsiYpi/1−YsYp	[157]	13.	RDI	YsiYpi÷YsYp	[158]
4.	TOL	$Y_{pi}-Y_{si}$	[152]	14.	DI	Ysi×YsiYpi÷Ysi	[159]
5.	GMP	$\sqrt{Y_{pi}\times Y_{si}}$	[91]	15.	GM	$\left(Y_{pi}+Y_{si}\right)/\left(Y_{pi}-Y_{si}\right)$	[160]
6.	REI	YsiYs×YpiYp	[155]	16.	ATI	$\left\{\frac{\left(Y_{pi}-Y_{si}\right)}{\left(Y_{p}÷Y_{s}\right)}\right\}\times \sqrt{Y_{pi}\times Y_{si}}$	[161]
7.	STI	$\left(Y_{si}\times Y_{pi}\right)/\left(P_{p}^{2}\right)$	[91]	17.	SSPI	$\left(\frac{Y_{pi}-Y_{si}}{2\times Y_{p}}\right)\times 100$	[161]
8.	MSTIk1	Ypi2Yp2×STI	[162]	18.	SNPI	$\sqrt[{3}]{\frac{Y_{pi}+Y_{si}}{Y_{pi}-Y_{si}}\times \sqrt[{3}]{Y_{pi}\times Y_{si}\times Y_{si}}}$	[161]
9.	MSTIk2	Ysi2Ys2×STI	[162]	19.	RDY	$100-\left\{\left(\frac{Y_{si}}{100}\right)\times Y_{pi}\right\}$	[163]
10.	HARM	2×Ypi×YsiYpi+Ysi	[164]	20.	DTE	$\left(Y_{si}/Y_{pi}\right)\times 100$	[165]

Ysi: yield under stress for genotype or treatment “i”; Ypi: yield under non-stress for genotype or treatment “i”; Ys: mean of yield under stressed; Yp: mean of yield under non-stress conditions. MP, mean productivity; MRP, mean relative performance; SSI, stress susceptibility index; TOL, tolerance index; GMP, geometric mean productivity; REI, relative efficiency index; STI, stress tolerance index; MSTIk1, modified stress tolerance index 1; MSTIk2, modified stress tolerance index 2; HARM, harmonic mean of yield; YI, yield index; Red, reduction; RDI, relative drought index; DI, drought resistance index; GM, golden mean; ATI, abiotic tolerance index; SSPI, stress susceptibility percentage index; SNPI, stress/non-stress production index; RDY, relative decrease in yield; DTE, drought tolerance efficiency. The index value indicates drought tolerance as high in the case of MP, MRP, GMP, REI, STI, MSTIk1, MSTIk2, HARM, YI, RDI, DI, GM, SNPI, and DTE and low in the case of SSI, TOL, Red, ATI, SSPI, and RDY.

**Table 8:** The energy equivalents of different inputs and outputs in maize production systems.

Equipment/Inputs	Unit	Energy Equivalent Coefficient (MJ/Unit)	Reference	Quantity per Hectare
A. Inputs				
1. Human labor	H	1.96	[142,167]	T0 = 378.9; T1, T3, T4, T6 = 402.9; T2, T5 = 426.9
2. Machinery	H	64.8	[168]	30.4
3. Nitrogen (N)	kg	66.14	[167,169]	194
4. Phosphorus (P)	kg	12.44	[167,169]	50
5. Potassium (K)	kg	11.15	[169]	50
6. Pesticides	kg	120	[142]	0.31
7. Plant growth regulator (PGR)	kg	85	[170]	T0 = 0.0; T1, T3 = 0.281; T2, T4 T6 = 0.562; T5 = 1.124
8. Diesel	L	56.31	[171]	524.2
9. Seed of maize	kg	14.7	[142]	25
B. Output				
1. Maize	kg	14.7	[167]	Treatment-wise grain yield

## Data Availability

The data presented in this study are available upon request from the corresponding authors.

## Supplementary Materials

The following supporting information can be downloaded at: https://www.mdpi.com/article/10.3390/plants12244192/s1, Figure S1: Yearly long-term climate during 1901–2021 at experimental location, Pakchong, Nakhon Ratchasima, Thailand. Yearly rainfall (total) in mm, and temperature (maximum, mean and minimum) in °C, Table S1: Analysis of variance for the effect of water levels, genotypes, and their interaction on different agro-physio-biochemical traits of maize in the greenhouse, Table S2: Analysis of variance for the effect of ethephon application tactics on different agronomic traits of maize under rainfed conditions at field level, Table S3: Analysis of variance for the effect of ethephon application tactics on different agro-physio-biochemical traits of maize under rainfed conditions at field level.
