# Supplementary material for: Potentiality of Sustainable Maize Production under Rainfed Conditions in the Tropics by Triggering Agro-Physio-Biochemical Traits Ascertained from a Greenhouse
Source: Plants (Basel). 2023 Dec 18;12(24):4192. doi: 10.3390/plants12244192 (PMC10748014; doi:10.3390/plants12244192)
Supplement: Supplementary file 1 [file plants-12-04192-s001.zip › plants-2712545-SI.pdf]

Supplementary Materials

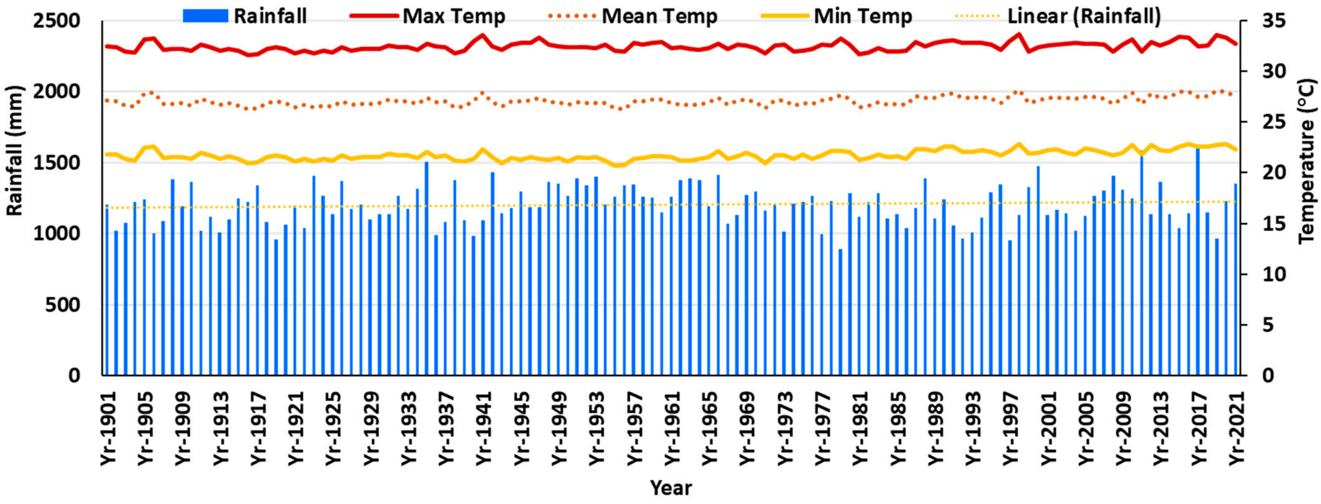

Figure S1. Yearly long-term climate during 1901-2021 at experimental location, Pakchong, Nakhon Ratchasima, Thailand. Yearly rainfall (total) in mm, and temperature (maximum, mean & minimum) in °C.

Table S1. Analysis of variance for the effect of water levels, genotypes, and their interaction on different agro-physio-biochemical traits of maize in the greenhouse.

Source of variance	df	Mean square																				
		RL3 (cm)	SBD3 (cm)	PH3 (cm)	LA1 (cm^2 $plant^{-1}$)	LA2 (cm^2 $plant^{-1}$)	LA3 (cm^2 $plant^{-1}$)	APB3 (g $plant^{-1}$)	RW3 (g $plant^{-1}$)	TPB1 (g $plant^{-1}$)	TPB2 (g $plant^{-1}$)	TPB3 (g $plant^{-1}$)	RGR-T1 (mg $plant^{-1}$ d^{-1})	RGR-T2 (mg $plant^{-1}$ d^{-1})	RGR-T3 (mg $plant^{-1}$ d^{-1})	NAR-T1 (mg cm^{-2} d^{-1})	NAR-T2 (mg cm^{-2} d^{-1})	NAR-T3 (mg cm^{-2} d^{-1})	SPAD3	RWC3 (%)	Pr3 (µmol g^{-1} FW)	TSS3 (mg g^{-1} FW)
Replication	3	11.31	3.41	17.00	25.99	49.57	216.3	1.65	0.23	0.52	1.67	3.13	47.84	13.43	28.42	1.28	0.28	0.47	19.05	86.97	0.01	0.02
Water level (W)	2	18.63**	29.54**	446.44**	2019.27**	1650.45**	10687.4**	152.04**	9.18**	24.78**	105.21**	235.20**	255.69**	198.54**	311.11**	10.54**	8.85**	3.58**	106.50**	827.58**	0.51**	0.54**
Genotype (G)	2	51.84**	26.52**	70.64**	317.17**	950.41**	1622.6**	16.96**	5.62**	1.30**	10.77**	39.26**	492.13**	279.91**	577.77**	1.92**	3.92**	4.13**	12.32*	511.23**	0.06**	0.17**
W×G	4	1.72ns	6.01**	13.79**	67.37**	73.91**	217.2**	1.94**	0.09*	0.73**	0.61ns	1.96*	33.59**	120.69**	111.11**	3.34**	5.40**	2.19**	3.37ns	95.10**	0.07**	0.06**

Source of variance	df	Mean square																				
		RL3 (cm)	SBD3 (cm)	PH3 (cm)	LA1 (cm^{2}plant^{-1})	LA2 (cm^{2}plant^{-1})	LA3 (cm^{2}plant^{-1})	APB3 (g plant^{-1})	RW3 (g plant^{-1})	TPB1 (g plant^{-1})	TPB2 (g plant^{-1})	TPB3 (g plant^{-1})	RGR-T1 (mg plant^{-1} d^{-1})	RGR-T2 (mg plant^{-1} d^{-1})	RGR-T3 (mg plant^{-1} d^{-1})	NAR-T1 (mg cm^{-2} d^{-1})	NAR-T2 (mg cm^{-2} d^{-1})	NAR-T3 (mg cm^{-2} d^{-1})	SPAD3	RWC3 (%)	Pr3 (µmol g^{-1} FW)	TSS3 (mg g^{-1} FW)
Error	24	1.73	0.50	2.47	4.09	7.54	32.8	0.25	0.03	0.08	0.26	0.48	7.23	1.92	4.05	0.19	0.04	0.06	2.75	12.46	0.002	0.03**

RL3: root length from 3rd (final) sampling at 13 days after watering treatment started (DAWTS); SBD3: stem base diameter at 13 DAWTS; PH3: plant height at 13 DAWTS; LA1, LA2 & LA3: fully expanded green leaf area at 5, 9 & 13 DAWTS respectively; APB3: above ground plant dry biomass at 13 DAWTS; RW3: root weight at 13 DAWTS; TPB1, TPB2, TPB3: total plant dry biomass at 5, 9 & 13 DAWTS respectively; RGR-T1, RGR-T2, RGR-T3: relative growth rate based on total plant biomass at 5-9, 9-13 & 5-13 DAWTS, respectively; NAR-T1, NAR-T2, NAR-T3: net assimilation rate based on total plant biomass at 5-9, 9-13 & 5-13 DAWTS, respectively; SPAD: SPAD value for leaf greenness at 13 DAWTS; RWC3: relative water content at 13 DAWTS; Pr3: proline at 13 DAWTS; TSS3: total soluble sugar at 13 DAWTS; * and ** Significant at 0.05 and 0.01 levels respectively; ns: nonsignificant; df: degree of freedom.

Table S2. Analysis of variance for the effect of ethephon application tactics on different agronomic traits of maize under rainfed conditions at field level.

Source of variance	df	Mean square														
		PH-PM (cm)	EH-PM (cm)	EH:PH ratio (%)	SBD-VT (mm)	LA-V6 (cm^2 plant^{-1})	LA-V10 (cm^2 plant^{-1})	LA-VT (cm^2 plant^{-1})	LA-R3 (cm^2 plant^{-1})	APB-V6 (g plant^{-1})	APB-V10 (g plant^{-1})	APB-VT (g plant^{-1})	APB-R3 (g plant^{-1})	KNP (no. plant^{-1})	100-KW (g)	KY (t ha^{-1})
Replication	3	97.20	15.99	4.41	1.58	4411.41	31362	99474	50939	0.62	5.34	76.75	256.34	324.59	1.57	0.06
Ethephon (E)	6	791.88**	787.86**	93.67**	0.52ns	1346.36ns	688925**	1894430**	1231297**	0.21ns	0.12ns	244.12*	745.34*	190.09ns	6.74**	0.84**
Error	18	96.22	14.39	4.01	1.59	4401.33	29462	91070	47677	0.62	5.39	79.64	253.70	333.44	1.66	0.06

V6: vegetative 6 leaves stage; V10: vegetative 10 leaves stage; VT: vegetative tasseling stage; R3: reproductive phase-3 of maize plant; PM: physiological maturity stage; PH-PM: plant height at PM; EH-PM: ear height at PM; EH:PH: ear height and plant height ratio; SBD-VT: stem base diameter at VT; LA-V6, LA-V10, LA-VT, and LA-R3: leaf area at V6, V10, VT, and R3, respectively; APB-V6, APB-V10, APB-VT, and APB-R3: above ground plant biomass at V6, V10, VT, and R3, respectively; KNP: kernel number per plant; 100-KW: 100-kernel weight; KY: kernel yield.

Table S3. Analysis of variance for the effect of ethephon application tactics on different agro-physio-biochemical traits of maize under rainfed conditions at field level.

Source of variance	df	Mean square																	
		SLW-VT (g m^{-2})	LP-PM (%)	RGRA-V6V10 (mg $plant^{-1}$ day^{-1})	RGRA-V10VT (mg $plant^{-1}$ day^{-1})	RGRA-V6R3 (mg $plant^{-1}$ day^{-1})	NARA-V6V10 (mg cm^{-2} day^{-1})	NARA-V10VT (mg cm^{-2} day^{-1})	NARA-V6R3 (mg cm^{-2} day^{-1})	Energy efficiency	AGDD (°C days)	HUE (kg ha^{-1} °C $days^{-1}$)	RWC-VT (%)	SPAD-VT	RSR-VTR3 (%)	EL-VT (%)	C absorption (t C ha^{-1})	TSS-VT (mg g^{-1} FW)	Pr-VT (μmol g^{-1} FW)
Replication	3	6.38	0.11	0.82	2.38	0.01	0.001	0.001	0.002	0.01	8140	0.02	21.52	4.67	0.01	0.07	0.08	6373	1.35
Ethephon (E)	6	69.51**	83.32**	1.94ns	2.56ns	1.21**	0.001ns	0.006**	0.013**	0.08**	5837ns	0.19**	23.67ns	5.16ns	0.05**	0.53**	1.17**	60365**	38.92**
Error	18	6.86	0.10	0.82	1.49	0.01	0.000	0.001	0.002	0.01	8056	0.02	22.27	4.75	0.01	0.07	0.08	6853	1.52

V6: vegetative 6 leaves stage; V10: vegetative 10 leaves stage; VT: vegetative tasseling stage; R3: reproductive phase-3 of maize plant; PM: physiological maturity stage; SLW-VT: specific leaf weight at VT; LP-PM: lodging percentage at PM; RGRA-V6V10, RGRA-V10VT, and RGRA-V6R3: relative growth rate based on above ground plant biomass during V6 to V10, V10 to VT, and V6 to R3, respectively; NARA-V6V10, NARA-V10VT, and NARA-V6R3: net assimilation rate based on above ground plant biomass during V6 to V10, V10 to VT, and V6 to R3, respectively; AGDD: accumulated growing degree days; HUE: heat use efficiency; RWC-VT: relative water content at VT; SPAD-VT: SPAD value of leaf greenness at VT; RSR-VTR3: relative senescence rate during VT to R3; EL-VT: electrolyte leakage at VT; C: carbon; TSS-VT: total soluble sugar at VT; Pr-VT: proline content at VT.
